# The apparent interferon resistance of transmitted HIV-1 is possibly a consequence of enhanced replicative fitness

**DOI:** 10.1371/journal.ppat.1010973

**Published:** 2022-11-18

**Authors:** Elena Sugrue, Arthur Wickenhagen, Nardus Mollentze, Muhamad Afiq Aziz, Vattipally B. Sreenu, Sven Truxa, Lily Tong, Ana da Silva Filipe, David L. Robertson, Joseph Hughes, Suzannah J. Rihn, Sam J. Wilson

**Affiliations:** 1 MRC-University of Glasgow Centre for Virus Research, University of Glasgow, Glasgow, United Kingdom; 2 School of Biodiversity, One Health & Veterinary Medicine, University of Glasgow, Glasgow, United Kingdom; 3 Institute of Biological Sciences, Faculty of Science, University of Malaya, Kuala Lumpur, Malaysia; 4 Division of Systems Immunology and Single Cell Biology, German Cancer Research Center, Heidelberg, Germany; Tel Aviv University, ISRAEL

## Abstract

HIV-1 transmission via sexual exposure is an inefficient process. When transmission does occur, newly infected individuals are colonized by the descendants of either a single virion or a very small number of establishing virions. These transmitted founder (TF) viruses are more interferon (IFN)-resistant than chronic control (CC) viruses present 6 months after transmission. To identify the specific molecular defences that make CC viruses more susceptible to the IFN-induced ‘antiviral state’, we established a single pair of fluorescent TF and CC viruses and used arrayed interferon-stimulated gene (ISG) expression screening to identify candidate antiviral effectors. However, we observed a relatively uniform ISG resistance of transmitted HIV-1, and this directed us to investigate possible underlying mechanisms. Simple simulations, where we varied a single parameter, illustrated that reduced growth rate could possibly underly apparent interferon sensitivity. To examine this possibility, we closely monitored *in vitro* propagation of a model TF/CC pair (closely matched in replicative fitness) over a targeted range of IFN concentrations. Fitting standard four-parameter logistic growth models, in which experimental variables were regressed against growth rate and carrying capacity, to our *in vitro* growth curves, further highlighted that small differences in replicative growth rates could recapitulate our *in vitro* observations. We reasoned that if growth rate underlies apparent interferon resistance, transmitted HIV-1 would be similarly resistant to any growth rate inhibitor. Accordingly, we show that two transmitted founder HIV-1 viruses are relatively resistant to antiretroviral drugs, while their matched chronic control viruses were more sensitive. We propose that, when present, the apparent IFN resistance of transmitted HIV-1 could possibly be explained by enhanced replicative fitness, as opposed to specific resistance to individual IFN-induced defences. However, further work is required to establish how generalisable this mechanism of relative IFN resistance might be.

## Author summary

HIV-1 sexual transmission is surprisingly inefficient. In the relatively unusual event of successful transmission, there is typically a severe genetic bottleneck, such that infection is established by just a single (or small number) of genetic variants, described as transmitted founder (TF) viruses. Such limited transmission is considered to arise from both physical and immunological barriers that restrict viruses, such as the interferon system. TF viruses have been described as more interferon resistant than viruses present in chronically infected individuals. Here we found that an IFN-resistant transmitted HIV-1 virus is uniformly resistant to multiple interferon-stimulated genes, which led us to investigate the underlying mechanism. Our characterisation of a single TF and chronic virus pair (closely matched in replicative fitness) revealed that small differences in replicative growth rates could possibly explain the broad interferon resistance frequently associated with transmitted HIV-1, although further studies are required to confirm this. Our study provides insight into how relatively small differences in growth rate could still be phenotypically significant, and that a nonspecific mechanism could possibly explain the interferon resistance of transmitted HIV-1.

## Introduction

The type I interferon (IFN) response is one of the first immune defences deployed against invading pathogens, including HIV-1 [1]. IFN signalling results in the expression of hundreds of IFN-stimulated genes (ISGs), many of which restrict virus replication, thereby creating an antiviral state [2,3]. Individual ISGs can form powerful barriers to the successful cross-species transmission of HIV-1 and related primate lentiviruses (reviewed in [4]). Moreover, in experimental settings, IFN stimulation protects against simian immunodeficiency virus (SIV) infection (with more challenges being required to initiate infection) [5]. Thus, it is possible that individual ISGs might constrain the transmission of HIV-1 between individuals.

HIV-1 sexual transmission is a surprisingly inefficient process [6], with >98% of sexual exposure events not resulting in transmission [7]. Thus, the virions in fluids from infected individuals are usually unable to establish a productive infection in a new host. In the unusual event of successful transmission, there is typically a severe genetic bottleneck, such that infection is established by just a single genetic variant (or a very small number of variants), described as the transmitted founder (TF) virus(es) [8–12]. Such limited transmission arises from both physical and immunological barriers that restrict viruses from the typically large and diverse population present in the donor from productively infecting target cells in a new host [13–15]. Understanding the molecular details that underlie successful transmission could substantially improve vaccine/prophylactic strategies and there is thus great interest in understanding the degree to which the transmitted variants are selected by chance or by specific phenotypic properties. This has led to extensive research on the phenotypic properties of transmitted HIV-1 [9,16–31]. However, only a small subset of the millions of successful transmission events that have occurred have been investigated phenotypically and agreement regarding the properties that might favour transmission has yet to emerge. Notably, TF viruses are usually CCR5-tropic (or CCR5/CXCR4 dual-tropic) [9,16–19] and have a tendency to be more consensus-like than the populations from which they are derived [21,32], although the strength of this signal can be variable [25]. Importantly, favourable transmission of variants with consensus-like signatures implies that there is phenotypic selection at the point of transmission.

Multiple studies have reported that TF viruses can be more IFN resistant than viruses isolated during chronic infection [18,20,31] or derived from the relevant donor [26]. Many ISGs are known to encode restriction factors that are capable of inhibiting HIV-1 (such as tripartite motif containing 5 (TRIM5) [33], apolipoprotein B mRNA editing enzyme catalytic subunit 3G (APOBEC3G) [34] and tetherin [35]), but HIV-1 either evades these factors or deploys countermeasures to ensure successful replication [33–36]. Although viral countermeasures to major restriction factors undoubtedly mitigate the antiviral effects of IFNs [37], chronic control (CC) viruses maintain their accessory genes over the course of infection [38] meaning that other factors likely underpin the observed relative IFN-resistance of transmitted HIV-1. Indeed, TF viruses have previously been observed to be relatively resistant to the IFITMs (interferon-induced transmembrane proteins), with this resistance reported to decrease during chronic infection and prolonged exposure to the host immune response [24]. Importantly, although multiple studies have implicated IFN-resistance as a key property of TF viruses, this is by no means universally observed [21,25]. This is perhaps unsurprising in light of the dynamic nature of IFN resistance during chronic infection, as the IFN-sensitivity of chronic/non-transmitted variants may be very different at different times following infection [31].

A mechanistic understanding of the specific molecular defences that make CC viruses more susceptible to the antiviral state is currently incomplete. Our initial aim was to use arrayed ISG expression screening to identify specific antiviral defences that disproportionately inhibit CC viruses and are resisted by TF viruses. We instead unexpectedly reveal the relatively uniform ISG resistance profile of a representative transmitted HIV-1. Our subsequent *in vitro* characterisation of a model TF/CC pair (closely matched in replicative fitness) was reminiscent of simple illustrative simulations indicating that reduced growth rate could underlie apparent IFN sensitivity. Moreover, fitting standard four-parameter logistic growth models to our *in vitro* data highlighted that small differences in replicative growth rates could possibly explain the broad IFN resistance displayed by transmitted HIV-1. These unanticipated observations suggest that small fitness advantages could be a possible explanation for the apparent IFN resistance of transmitted HIV-1.

## Results

### Transmitted HIV-1 is more resistant to IFN

Identifying specific molecular defences that explain the relative resistance of HIV-1 transmitted founder (TF) viruses to IFN, when compared to matched CC viruses present 6 months after transmission, first required selection of an appropriate matched TF/CC pair for screening experiments. To select a pair, we examined the replication of four previously described infectious molecular clone (IMC) pairs [20] in immortalized human T cells over several days. Notably, each pair is representative of one infected individual, and the CC virus and TF virus are both derived from the infected recipient (i.e., they are not derived from transmission pairs/donor and recipient). To start the initial infections, which all used equivalent 0.01 multiplicities of infection (MOIs), we used virions pseudotyped with the vesicular stomatitis virus glycoprotein (VSV-G) in order to circumvent the low levels of infection typically observed with some IMCs. Specifically, to make infectious virus, HEK 293Ts were co-transfected with each IMC and a plasmid expressing the VSV glycoprotein (VSV-G), generating virions decorated with VSV-G and the relevant HIV-1 glycoprotein. Crucially, the IMCs were not genetically modified, so after the first round of infection, entry was mediated by the HIV-1 envelope (encoded by the proviral genome).

**Fig 1:**
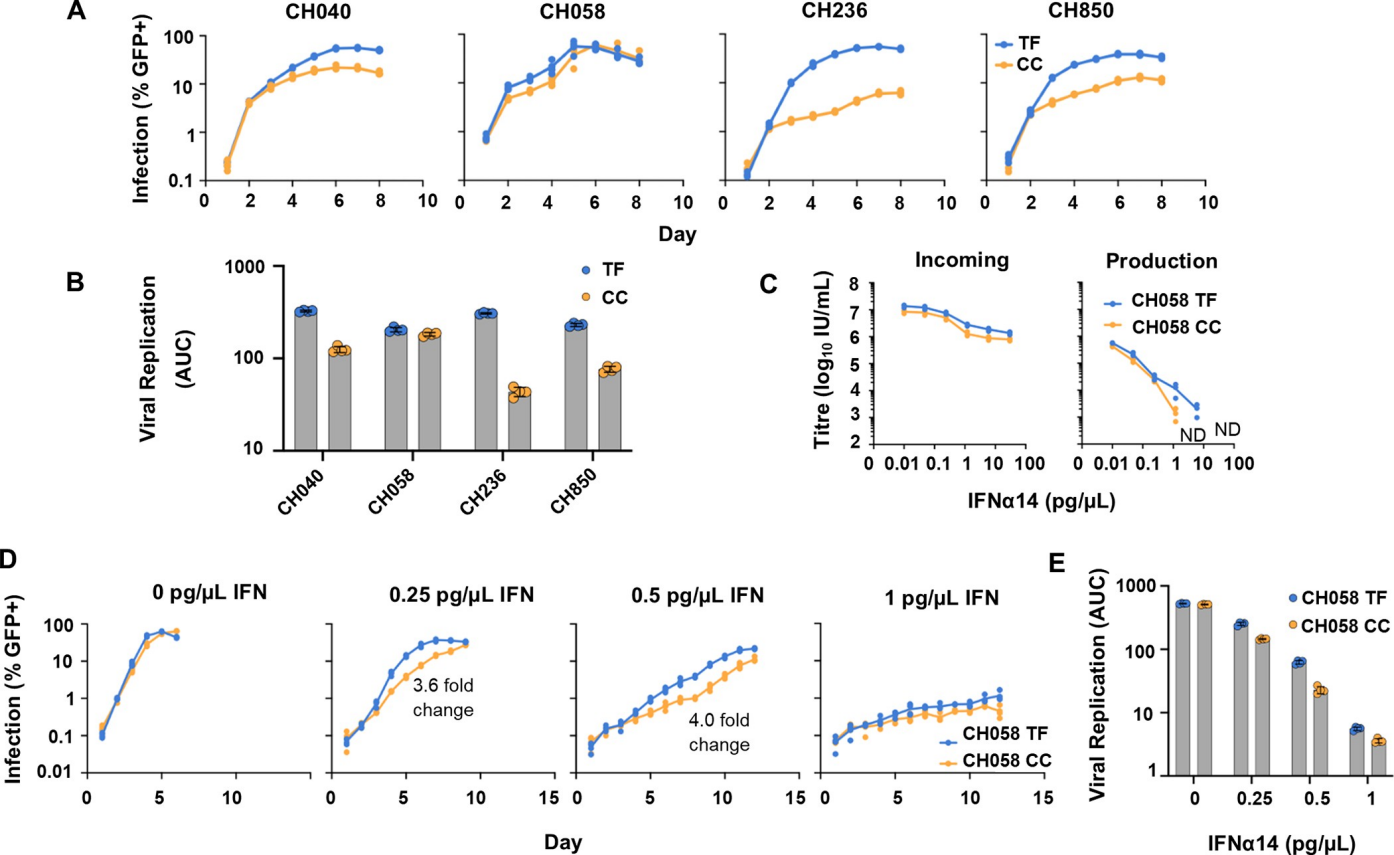
The CH058 TF virus is more resistant to IFN than its matched CC virus. (A-B) TMZR5 GFP-reporter cells were challenged with matched TF and CC VSV-G pseudotyped IMC pairs and sampled daily to monitor virus spread. GFP-positive cells were enumerated via flow cytometry. (C) To investigate the effect of IFN early in the viral life cycle (incoming infection), TMZR5 cells were pre-stimulated with different doses of IFNα14 for 24 hours prior to infection with serially diluted CH058 TF and CC viruses. To limit replication to a single cycle, infected cells were treated with dextran sulphate 17–18 hours post infection. The percentage of GFP-positive cells was determined at 48 hours post-infection using flow cytometry. To investigate the effect of IFN late in the viral life cycle (production effect), TMZR5 cells were pre-treated with IFNα14, and after 24 hours cells were challenged with CH058 TF and CC viruses at an MOI of 0.5 for 6 hours, before the inoculum was removed. At 46–48 hours post-infection, cell-free, filtered virus containing supernatants were titrated on TMZR5 cells. ND indicates not detected. (D-E) TMZR5s were treated with the indicated dose of IFNα14 for 24 hours before being challenged with the CH058 TF and CC virus pair. Cells were sampled daily to monitor virus spread and GFP-positive cells were enumerated via flow cytometry. Annotated fold change values refer to the maximum difference in (%) infection out of the timepoints tested. Viral spreading replication experiments took place on two occasions and a typical result is shown.

To visualise the spread of the unmodified viruses in subsequent rounds of infection, we used an LTR-GFP reporter cell line, MT4 TMZR5 cells [39], which fluoresces green when infected and which could be monitored daily via flow cytometry. Notably, three of the pairs tested (CH040, CH236 and CH850) exhibited large differences in replicative fitness in the absence of IFN, which would make examining the relative resistance of these pairs to IFN challenging (Fig 1A and 1B). In contrast, the CH058 TF/CC pair exhibited similar replicative kinetics in the absence of IFN, as well as high levels of overall infection (Fig 1A and 1B). Therefore, the CH058 pair [17,20,40] was selected for use as a model pair for our subsequent screening experiments. Additionally, to remove any confounding issues from pseudotyping with VSV-G, the CH058 IMC pair was instead propagated in TMZR5 cells (using stocks produced without pseudotyping) to generate sequence-verified working stocks for subsequent experiments.

ISGs induced by type I IFNs can confer protection against HIV-1 either during early (incoming) infection [33,34,41–43], or can inhibit the production of infectious progeny [35,44]. Pre-treatment of TMZR5 cells with varying concentrations of IFN⍺14 stimulated modest ~10-fold protection against incoming infection from the CH058 TF and CC viruses tested (Fig 1C). At doses lower than 0.24 pg/μl, the incoming titres of both TF and CC viruses were unaffected. As the concentration of IFN⍺14 increased above 0.24 pg/μl, the infectivity of both TF and CC viruses was moderately suppressed. We subsequently determined the infectious yields of CH058 TF and CC viruses using TMZR5 cells stimulated with varying concentrations of IFN⍺14 (Fig 1C). Without IFN stimulation, both viruses produced a similar level of infectious progeny virions. Elevating the dose of IFN⍺14 caused a substantial reduction in the infectious production of both CH058 viruses. Strikingly, at 6.0 pg/μl and higher, the infectious yield of the CH058 CC virus was reduced to below the level of detection, whereas infectious CH058 TF was readily detectable (Fig 1C). This indicates that IFN⍺14 caused a stronger reduction in the infectious yield of the CC virus than of the TF virus (using the CH058 pair), and also suggests that IFN⍺14 confers a relatively weak early block (~10-fold) and potent late block (>200-fold) to HIV-1 CH058 in TMZR5 cells. Using our culture conditions, we did not observe an IFN-dependent enhancement of cell-free CH058 infection that has been proposed for CH077 (under specific conditions) [45].

To further investigate the impact of IFN⍺14 on CH058 replication, we examined the ongoing replication (over a longer timescale) of the CH058 pair in cells pre-treated with a range of IFN⍺14 doses. Notably, the TF virus again outperformed the CC virus across all the IFN doses tested, despite comparable replication kinetics in the absence of IFN (Fig 1D–1E). Because of the proapoptotic effect of IFNs, the viability of IFN-treated cells was also assessed in parallel cultures. The majority of IFN doses tested exhibited a live population of 80–90%, with the highest dose tested (1 pg/ μl) displaying a ~60% live population (S1 Fig).

### ISG expression screening reveals multiple ISGs that inhibit the CC virus more potently than transmitted HIV-1

**Fig 2:**
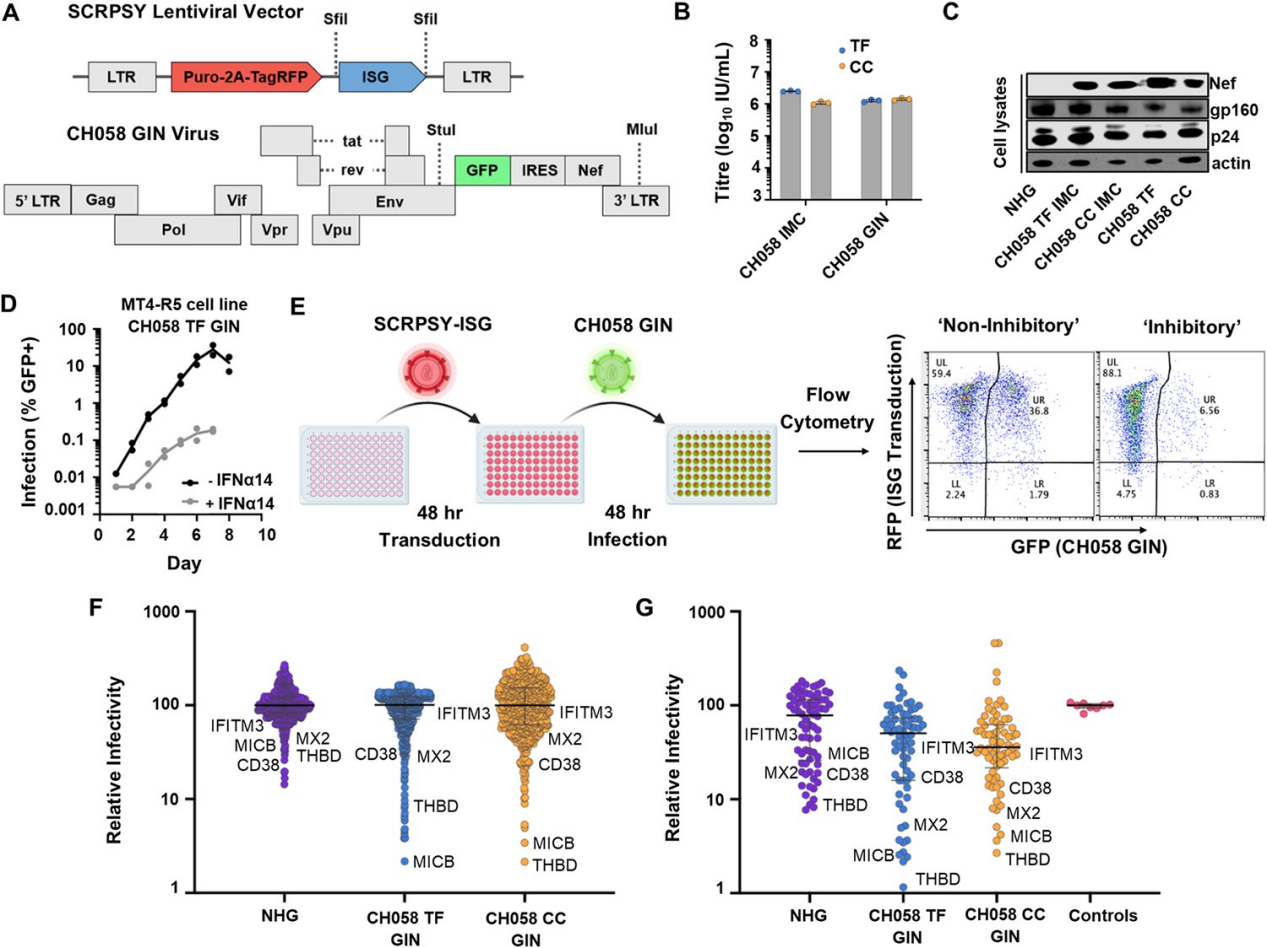
Arrayed ISG expression screening identifies multiple ISGs that inhibit transmitted HIV-1 and its matched CC virus. (A) A schematic of the SCRPSY lentiviral vector (GenBank accession KT368137.1) used to deliver ISGs, and of the fluorescent GFP-IRES-Nef (GIN) TF and CC viruses used. (B) The infectious titres of CH058 IMC and CH058 GIN viruses in a single cycle of infection in TMZR5 cells. Cells were treated with dextran sulphate 17–18 hours post infection and the percentage of GFP positive cells was determined at 48 hours post-infection using flow cytometry. (C) Western blot analysis of viral antigens present in TMZR5 cell lysates infected with the indicated virus for 48 hours. (D) Growth kinetics of CH058 TF GIN in clonal MT4 CCR5-R126N in the absence and presence of 0.5 pg/μl IFNα14. Cells were treated with IFNα14 24h prior to infection. (E) A schematic of the ISG screening pipeline used in panel F. (F) Normalized infection (median centred) of cells expressing different ISGs (each dot represents the observed infection in the presence of a single ISG). (G) Validation screen, conducted as in E and F, of ISGs ‘hits’ selected that were more inhibitory than human Mx2 in panel F. Empty SCRPSY transduced MT4-R5 cells were used as controls.

We have previously used arrayed ISG expression screening to identify antiviral factors targeting a range of viruses [2,46–48]. Although HIV-1 has previously undergone large-scale ISG and CRISPR screening [2,3,49], a matched TF/CC pair has not yet been investigated in this way and could reveal specific molecular defences resisted by transmitted HIV-1. We therefore conducted ISG screening using our human ISG library, which includes >500 unique ISGs encoded in SCRPSY lentiviral vectors (Fig 2A), in conjunction with a GFP-encoding TF/CC pair (CH058) we developed in order to enable easy quantification of virus infection using flow cytometry. To construct this GFP TF/CC pair, we inserted an internal ribosome entry site (IRES)-GFP cassette between *env* and *nef*, to create the viruses we refer to as the CH058 GIN (GFP-IRES-*nef*) viruses (Fig 2A).

As described above, the CH058 pair was chosen as an ideal pair for screening because its viruses exhibited the most similar replication kinetics (Fig 1A and 1B). Stocks of the GIN viruses were prepared (Fig 2B) and viral protein expression (Fig 2C) was assessed and was found to be comparable to the unmodified CH058 IMCs. We then elected to conduct the ISG screens in MT4 cells modified to express a signalling-defective variant of CCR5 [50]. Importantly, these cells, referred to as MT4-R5 cells, are both readily transduced by our ISG library, and also support efficient HIV-1 replication that is potently inhibited by type I IFN treatment (Fig 2D). We transduced the MT4-R5 cells with the ISG-encoding lentiviral library and, 48 h later, infected these cells with CH058 TF GIN and CH058 CC GIN viruses (Fig 2E). At 96 h post-infection, the level of CH058 GIN infection in the presence of each individual ISG was quantified using flow cytometry (Fig 2E and 2F).

Due to the low levels of infection that would occur in a single replication cycle from the GIN variants of CH058, we assessed multi-cycle infection in the ISG screens for these viruses. However, as these multi-cycle infections could mask potential anti-HIV-1 genes acting early in the life cycle, we also conducted a single-cycle ISG screen using lab-adapted HIV-1 NHG (Fig 2F), which is an NL4.3-derived virus, that contains portions of the HxB envelope, and that encodes GFP in place of *nef* [51]. Following completion of these screens, and in order to pinpoint specific ISGs that inhibit HIV-1, we identified all genes that showed equivalent or stronger inhibition than the known anti-HIV-1 ISG Mx2 in any individual screen [52]. We then subtracted known IFNβ/ISRE-inducing ISGs [2] from this list and re-examined the ability of independent lentiviral vector preparations encoding each of these potentially antiretroviral ISGs to inhibit HIV-1 (Fig 2G). Following this ‘miniscreen’, we selected all the ISGs (25 candidate genes) that exhibited inhibition equivalent or stronger than that displayed by IFITM3, an ISG resisted by transmitted HIV-1 [24], for subsequent analysis.

**Fig 3:**
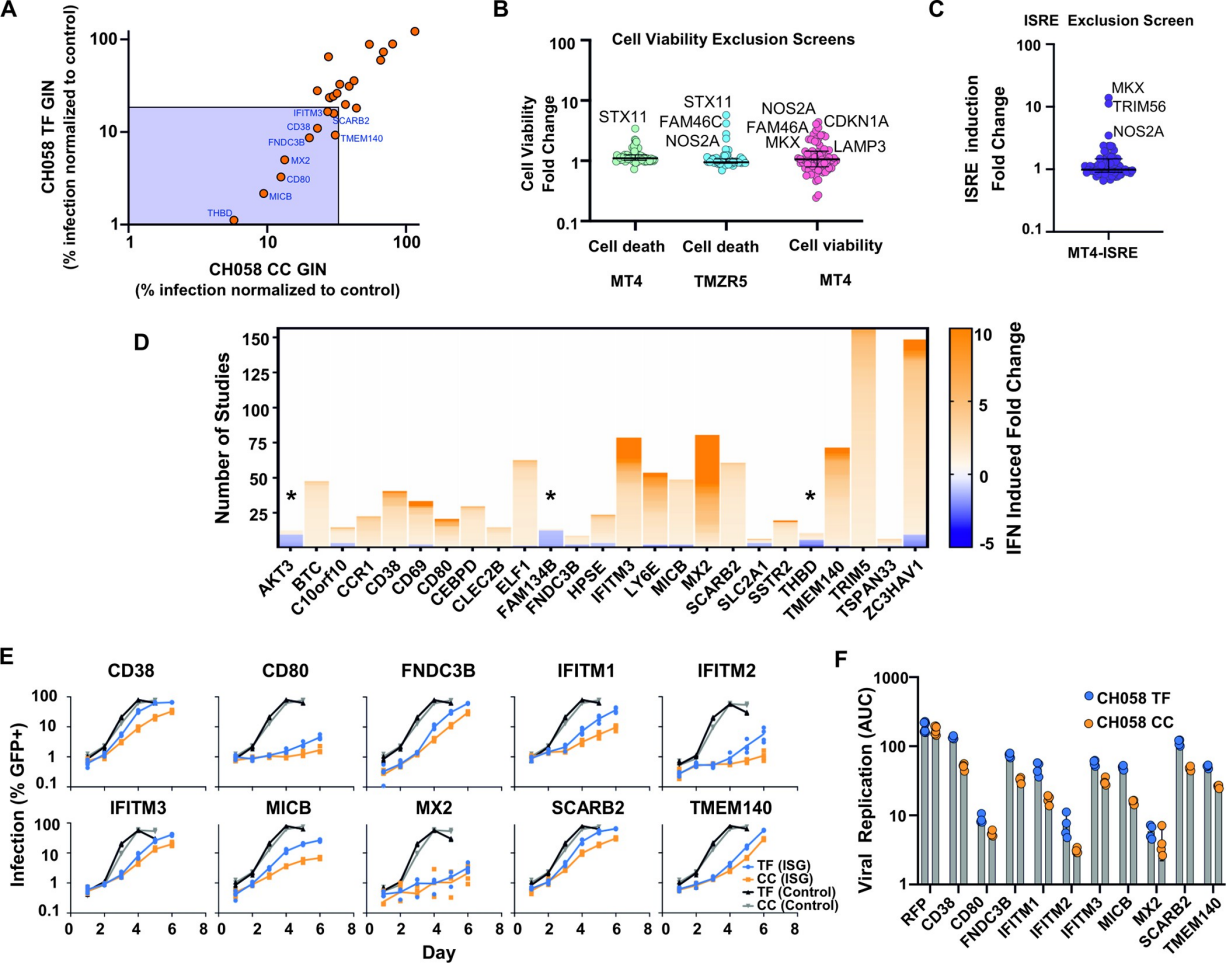
Anti-HIV-1 ISGs inhibit the CC virus more potently than transmitted HIV-1. (A) Candidate anti-HIV-1 effectors that were more inhibitory than the known anti-HIV-1 effector IFITM3 were tested against CH058 GIN GFP-IRES-Nef) TF and CC viruses on MT4-R5 cells, as in 2F-G, normalised to the level of infection observed in the presence of an empty vector control. (B-C) ISGs in Fig 3A were tested for ability to induce cell death or ISRE (IFN-stimulated response element). In B, MT4 and TMZR5 cells were tested for cell death when expressing ISGs by flow cytometry using the LIVE/DEAD fixable dead cell stain kit (Invitrogen) and MT4 cell viability was additionally tested using the luminescence based CytoTox-Glo Cytotoxicity assay (Promega). In C, IFN induction by candidate ISGs was measured by flow cytometry using MT4 cells expressing an ISRE-GFP construct. (D) Candidate ISGs from Fig 3A were checked for their changes in expression upon type I IFN stimulation using the Interferome database to determine their ‘ISG-ness’. (E-F) Validation of the 8 most potent anti-HIV-1 effector ISGs, along with IFITM1 and IFITM2 controls, against CH058 TF and CC viruses, In E, TMZR5 cells transduced with pLV constructs containing the indicated ISGs or RFP as a control, were challenged with CH058 TF or CC and sampled daily to monitor virus spread. GFP-positive cells were enumerated using flow cytometry. Viral spreading replication experiments took place on two occasions, a typical result with contemporaneous controls is shown. In F, data from panel E represented as area under the curve (AUC).

We next examined the ability of these 25 candidate anti-HIV-1 effectors, and an empty vector control, to inhibit CH058 TF GIN and CH058 CC GIN HIV-1 in a multi-cycle infection on MT4-R5 cells transduced with an independent batch of lentiviral vectors expressing each candidate effector (Fig 3A). To potentially exclude genes from our final selection that are either ISRE- or cell death-inducing, we conducted four subtractive screens using our 25 candidate effector genes identified from Fig 2G. We tested the ability of all these genes to induce cell death in MT4 or TMZR5 cells (Fig 3B), examined the cell viability of MT4 cells transduced with these genes (Fig 3B) and assessed ISRE stimulation in an MT4-ISRE-GFP cell line transduced with these genes (Fig 3C). Genes showing more than 2.1-fold increase in any of these screens were excluded from further analysis. Additionally, we used published studies from the Interferome v2.0 database [53] to investigate the ‘ISG-ness’, or degree to which a gene is stimulated by interferon (Fig 3D). This led us to exclude AKT serine/threonine kinase 3 (AKT3), family with sequence similarity 134 member B (FAM134B) and thrombomodulin (THBD), as their type I IFN stimulation profile showed downregulation in more than half the published datasets where differential expression was observed (Fig 3D). Based on their strong anti-HIV-1 activity (Fig 3A), no considerable induction of cell death or ISRE stimulation (Fig 3B–3C), and strong IFN-stimulation (Fig 3D), we selected CD38, CD80, fibronectin type III domain containing 3B (FNDC3B), interferon induced transmembrane protein 3 (IFITM3), MHC class I polypeptide-related sequence B (MICB), MX dynamin like GTPase 2 (Mx2), scavenger receptor class B member 2 (SCARB2) and transmembrane protein 140 (TMEM140) as the final 8 candidate genes which exhibited strong anti-HIV-1 activity in our screens. Mx2 and IFITM3 are ISGs known to target HIV-1 [24,41,42,52,54,55], whereas the other genes have not yet been intensively investigated with regards to anti-HIV-1 activity.

The final candidate anti-HIV-1 effectors, alongside IFITM1 and IFITM2 controls [24], were then subcloned into a pLV lentiviral expression vector, and were used to stably modify GFP-reporter TMZR5 cells [39] to generate a cell line expressing each ISG. These cells were then infected with a low MOI (0.01) of unmodified CH058 TF and CH058 CC virus and the cultures were sampled daily to monitor virus spread (Fig 3E and 3F). All 10 exogenously expressed genes robustly inhibited HIV-1 replication when compared to an RFP control. Yet strikingly, comparisons of the CC and TF CH058 virus results revealed that the transmitted variant of CH058 was relatively resistant to all the ISGs tested except Mx2.

Given that six of the genes identified using our pipeline (CD38, CD80, FNDC3B, MICB, SCARB2 and TMEM140) have not been characterised as encoding anti-HIV-1 effectors, we wanted to further investigate the role endogenous expression of these ISGs could play in the anti-HIV-1 effects of IFN. We thus used western blotting to screen the endogenous expression levels of all six ISGs in a variety of cell lines and primary cells, in the presence and absence of IFN, in order to detect IFN-induced expression, and to also identify the best targets for CRISPR/Cas9 manipulations (S2 Fig). Analysis of these western blots identified both CD38 and SCARB2 as potential endogenous effectors, as both exhibited readily detectable endogenous expression, with observable increases in the presence of IFN. In contrast, the endogenous expression of CD80, FNDC3B and MICB was only weakly IFN inducible, and levels were considerably lower than the exogenous levels that inhibited HIV-1 in Fig 3E (S2 Fig). In addition, we were unable to convincingly detect TMEM140 expression using western blotting.

To investigate whether endogenous SCARB2 and CD38 might inhibit HIV-1, we disrupted these loci using CRISPR/Cas9. We examined the protein expression of each target using transduced ‘bulk’ populations and identified guides that reduced CD38 expression in PM1 cells, as well as guides that attenuated SCARB2 expression in TMZR5 cells (S2 Fig). We also examined HIV-1 replication in the two cell lines with the greatest reduction in endogenous expression (of CD38 or SCARB2) and observed no notable changes in HIV-1 replication compared to the non-targeting control cell lines (in the presence and absence of IFN). This suggests CD38 and SCARB2 are unlikely to play a major role in the inhibition of HIV-1 by type I IFNs *in vivo* (S2 Fig).

### Small differences in either growth rate between a virus pair, or in resistance to inhibition, are amplified by logistic growth

Because we had designed our study to identify specific effectors resisted by transmitted HIV-1, we did not anticipate that the TF would be relatively resistant to nearly all the candidate antiviral effectors tested (Fig 3E). Moreover, because ISGs like CD38 and SCARB2 are unlikely to inhibit HIV-1 in natural settings, it is similarly unlikely that HIV-1 would be selected to specifically resist these factors. Therefore, it seemed likely that the TF virus resisted the candidate anti-HIV-1 ISGs through a common, nonspecific mechanism. Notably, a previous study of HIV-1 transmission, that did not detect consistent IFN-resistance in transmitted variants, nonetheless observed a strong correlation between replicative fitness *in vitro* and interferon resistance (when multiple transmitted and non-transmitted variants in the study were considered) [21].

**Fig 4:**
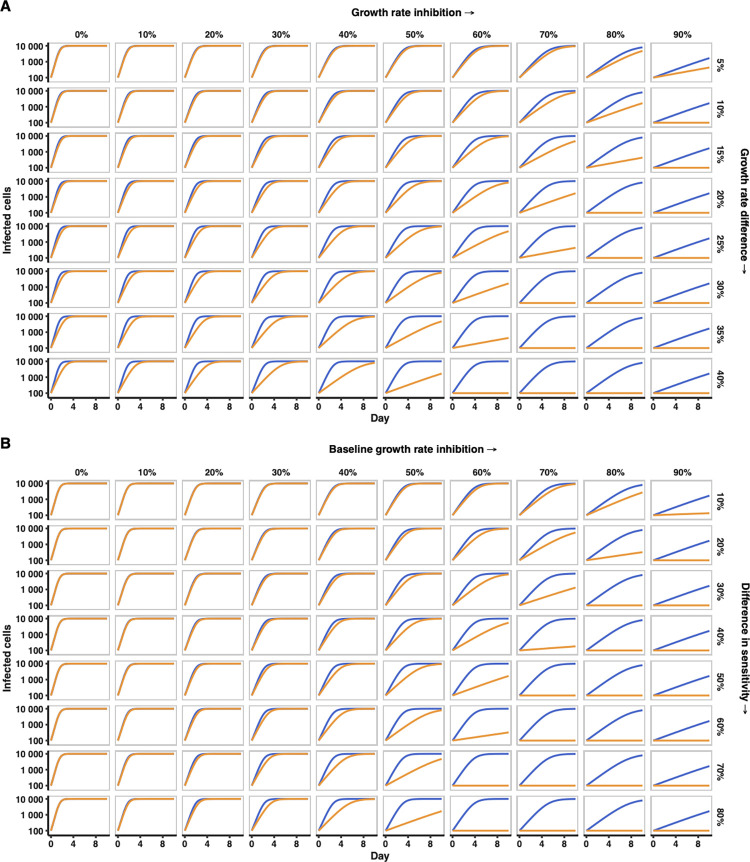
Illustrative simulations (in which a single parameter is varied) suggest that when assuming a simple logistic growth process, both small differences in growth rate between a virus pair, and differences in sensitivity to growth inhibition, can result in growth curves reminiscent of the relative interferon resistance of transmitted HIV-1 (observed in viral propagation experiments, such as those in Fig 1). (A) Illustrative logistic growth simulation of two viruses, where the growth rate of virus two (orange) is scaled relative to that of virus one (blue), while all other logistic growth parameters remain constant. Rows represent an increasing difference in growth rates between viruses (i.e., an increasing difference in replicative fitness). Both viruses experience the same relative growth rate inhibition (columns) mimicking increasing IFN stimulation. (B) Illustrative logistic simulations in which growth rates are identical, but virus two (orange) is more sensitive to the growth rate-inhibiting factor (i.e., one virus is more sensitive to antiviral effectors). Columns represent an increasing growth rate inhibition (mimicking increasing IFN stimulation), while rows represent an increasing difference in the sensitivity of the two viruses to that inhibition. As in (A), we assume that only growth rate is affected, with all other logistic growth parameters remaining constant between viruses.

These observations led us to hypothesize that the apparent difference in IFN sensitivity between TF and CC viruses was not driven by resistance to specific antiviral defences but was instead a consequence of different virus growth rates of the TF and CC viruses (i.e., differences in replicative fitness as opposed to genetic resistance to specific effectors with anti-HIV-1 activity). We reasoned that under a standard logistic growth model, in which viruses undergo an initial lag phase followed by a period of exponential growth that is ultimately curtailed by limited resource availability (in this case the supply of target cells available for infection), small differences in growth rate would become accentuated by IFN. To illustrate this effect, we simulated growth curves for two viruses using a simple logistic growth model (as has previously been used to explain the dynamics of HIV-1 replication [56,57]), varying a single parameter (growth rate) either consistently (i.e., one virus always grows faster than the other) or as a function of IFN dose (i.e., the growth rate of one virus decreases faster with increasing dose than the other). All other parameter values (initial population size, carrying capacity, and timescale) broadly matched the conditions in our experiments (see Methods). As expected, these simple simulations illustrated that small differences in growth rate was one mechanism that recapitulated the apparent IFN resistance observed in our experimental data (Figs 4A, 1A, 1D and 3E), with the faster-growing virus entering the exponential phase at a time when the slower-growing virus is still in the lag phase. Under normal culture conditions in the absence of IFN (using MOIs typical of these experiments), the lag phase is largely by-passed due to the relatively rapid growth rate of HIV-1. In contrast, inhibiting the growth rate of both viruses by the same amount (simulating IFN stimulation with no difference in IFN-sensitivity), slowed growth to the point where the lag phase became observable (Figs 4A and S3). Notably, even a 10% difference in growth rates led to the faster-growing virus infecting ~10-fold more cells by day 4 in the presence of high concentrations of IFN (Figs 4A and 1A). However, visually similar dynamics could also be produced by assuming identical growth rates but a difference in inhibitor sensitivity (mimicking differing IFN sensitivity, specifically the CC virus being more sensitive to specific ISGs [24]), as this would result in an effective difference in growth rates whenever the inhibitor is present (Fig 4B).

**Fig 5:**
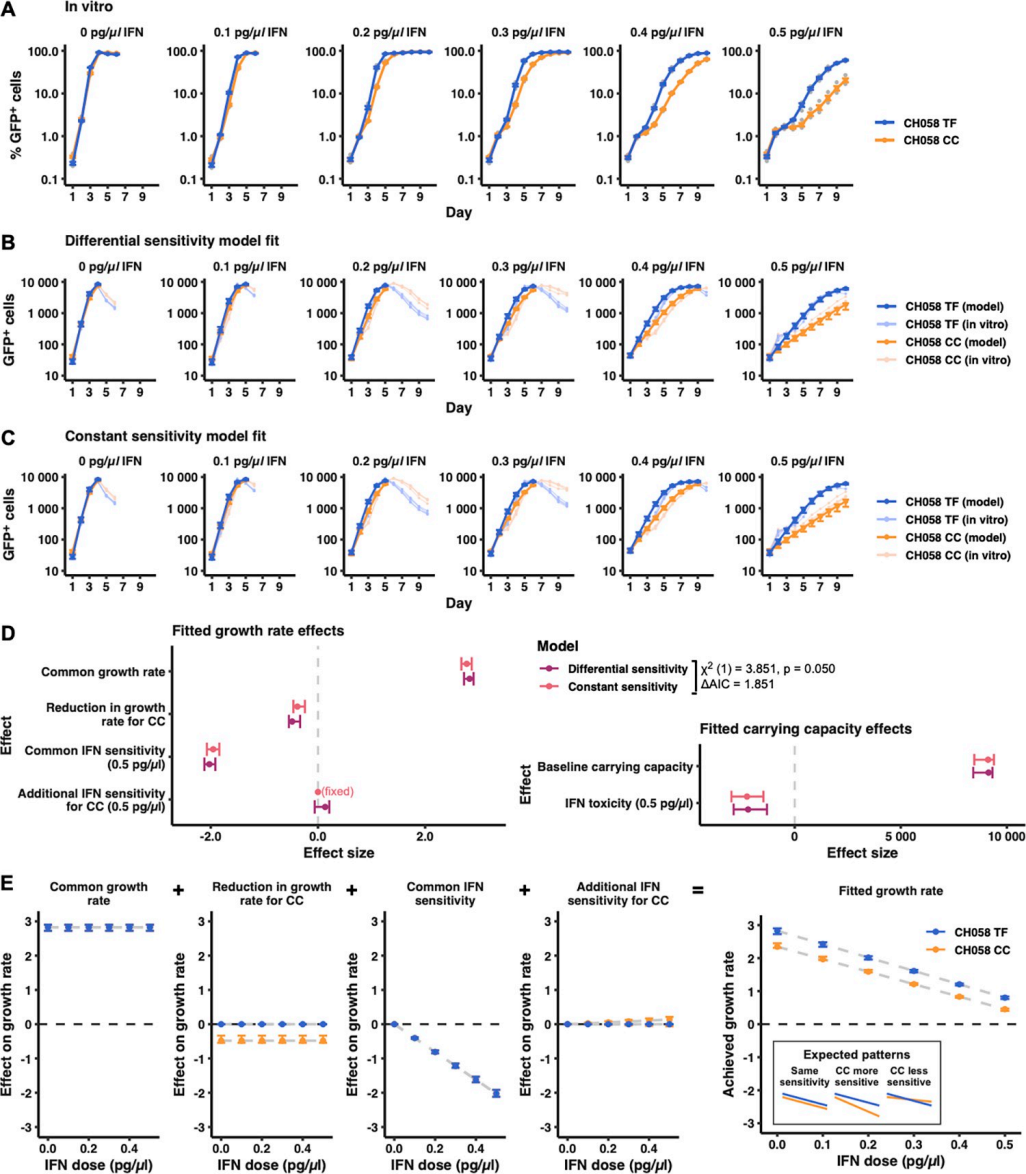
Statistical modelling of virus growth curves indicates that CH058 CC has a lower growth rate than its TF progenitor but is not otherwise more sensitive to IFN. (A) TMZR5 cells were pre-stimulated with different doses of IFNα14 for 24 hours prior to infection with CH058 TF or CC virus and sampled daily to monitor virus spread. GFP-positive cells were enumerated using flow cytometry. Blue and orange points show means (+/- standard error) across 4 experimental replicates, while translucent points show individual observations. Viral spreading replication experiments took place on two occasions, a typical result is shown. (B) Model fit for the differential sensitivity model, in which viruses are allowed to vary in both baseline growth rate (i.e., growth rate in the absence of interferon) and in their sensitivity to interferon. (C) Model fit for the constant sensitivity model, in which viruses differ only in their baseline growth rate. In both B and C, opaque points show predictions from the fitted model, along with 95% confidence intervals. Translucent points and lines underneath show the observed (experimental) replicate growth curves also present in A. For each treatment, only the initial timepoints until at least one replicate curve decreased by >30% relative to its preceding observation were used in model fitting (i.e. points after HIV-1 had overwhelmed the culture were not considered). (D) Effect sizes for both differential and constant sensitivity models (maximum likelihood estimates and 95% confidence intervals). Model fit was compared by likelihood ratio test and AIC, with results shown in the legend. (E) Illustration of effects making up the fitted growth rates in the differential sensitivity model. Effects were modelled as additive, allowing us to separate discrete contributions to the growth rates needed to recapitulate the *in vitro* data as illustrated in B. Points show maximum likelihood estimates, while error bars show 95% confidence intervals. An inset in the final panel (right) illustrates expected patterns under different hypothesized scenarios; in particular, if the CC virus was more sensitive to IFN, its achieved growth rate would have diverged from that of the TF virus at increasing IFN doses.

We therefore investigated the relative support for these contrasting hypotheses, i.e., whether the observed TF/CC growth kinetics were best explained by: (a) differences in TF/CC viral growth rates, (b) differences in their sensitivity to IFN inhibition, or (c) a combination of both differences in growth rate and in IFN sensitivity. To do this, we implemented a viral spreading assay using the CH058 TF/CC pair over a finely tuned range of IFN⍺14 doses, with a focus on increments between 0 and 0.5 pg/μl, as this is where the largest difference in replication was observed (Fig 1D). TMZR5 cells were pre-treated with IFN for 24 hours prior to virus inoculation, and the infection levels were monitored daily via flow cytometry (Fig 5A). We next fitted two alternative standard four-parameter logistic growth-based regression models to the observed number of infected (GFP+) cells (Fig 5B–5C). These models incorporated regressions on the growth rate and carrying capacity parameters (with the latter used to account for IFN toxicity, which had the effect of reducing the number of cells available to infection at higher doses of IFN). The first model (‘differential sensitivity model’, Fig 5B), allowed for differences in both growth rate and in IFN-sensitivity between the TF and CC viruses. This was contrasted with a simpler model, in which TF and CC viruses differed in growth rate only (‘constant sensitivity model’, Fig 5C). Both models were able to closely match the patterns seen in our experimental observations (Fig 5B and 5C), and we detected no meaningful difference in model fit (ΔAIC = 1.81, likelihood ratio test p-value = 0.05). Indeed, both models utilised reduced baseline growth rate of the CC virus to achieve optimal fitting (Fig 5D) and increased IFN-sensitivity of the CC virus went largely unused when available (Fig 5D–5F). In the differential sensitivity model, this meant that the difference between the fitted growth rates of TF and CC viruses did not change over the range of IFN doses, suggesting that neither virus was substantially more or less sensitive to IFN (Fig 5E). Instead, the constant ~17% lower growth rate of the CC virus (95% confidence interval: 12.1–18.8% lower) (Fig 5D–5E), was sufficient to recapitulate the apparent IFN resistance of transmitted HIV-1. Thus, modest increases in the replicative fitness of TF viruses is a possible underlying mechanism for the apparent interferon resistance of transmitted HIV-1.

### Transmitted HIV-1 is more resistant to both IFN and antiretroviral compounds

**Fig 6:**
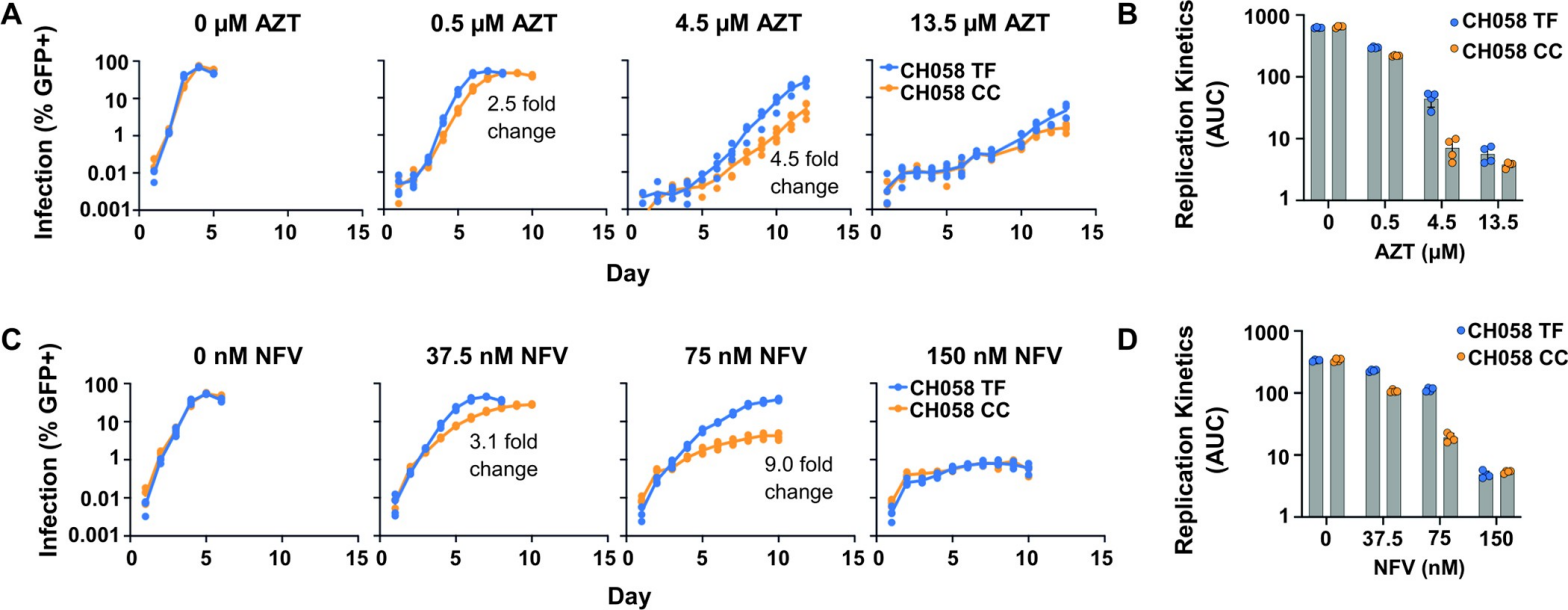
Transmitted HIV-1 is more resistant to antiretroviral drugs compared to the matched chronic virus. (A-B) TMZR5 cells were pre-treated with a range of azidothymidine (AZT) doses for 2 hours prior to infection with the CH058 TF or CC virus, cells were sampled daily to monitor virus spread. GFP-positive cells were quantified via flow cytometry. (C-D) TMZR5 cells were pre-treated with a range of nelfinavir (NFV) doses and infected, sampled and quantified as in A. Viral spreading replication experiments took place on two occasions, a typical result is shown.

**Fig 7:**
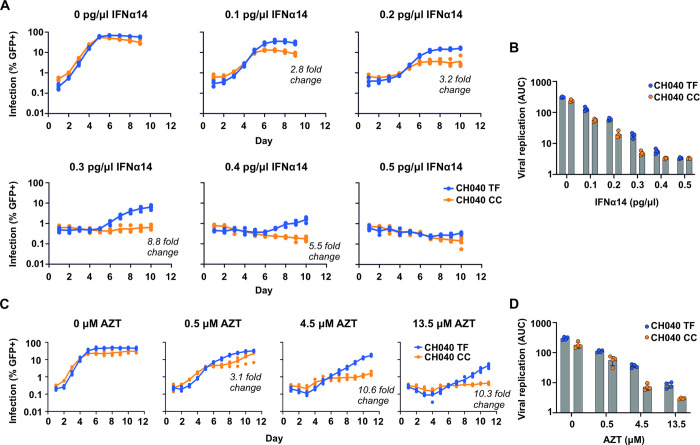
Transmitted HIV-1 of the CH040 virus pair is more resistant to IFN and AZT than the matched chronic virus. (A-B) TMZR5 cells were treated with indicated doses of IFNα14 for 24 hours before being challenged with the CH040 TF and CC viruses. Cells were sampled daily to monitor virus spread and GFP-positive cells were enumerated via flow cytometry. (C-D) TMZR5 cells were pre-treated with a range of azidothymidine (AZT) doses for 2 hours prior to infection with the CH040 TF or CC viruses. Cells were sampled daily to monitor virus spread and GFP-positive cells were quantified via flow cytometry.

If the apparent increased IFN resistance of transmitted HIV-1 is simply a by-product of enhanced replicative fitness, we hypothesized that transmitted HIV-1 would also be more resistant to other inhibitory agents, including those not normally encountered during sexual transmission. Notably, a similar relationship between replicative fitness conferred by *gag* and resistance to protease inhibitors has been suggested in a previous study [23]. We therefore investigated whether transmitted CH058 HIV-1 was more resistant to antiretroviral compounds. Crucially, we selected two antiretroviral compounds that would target viral proteins that were identical in the model TF and CC virus pair. There are only 8 amino acid differences between the TF and CC CH058 IMCs (*gag*: G251E, *tat*: K29R, *env*: T232A, N338D, R579S, A830T, *rev*: R54Q, *nef*: G113E, [20]) and the viruses encode identical protease and reverse transcriptase (RT) enzymes. Therefore, we considered the ability of the RT inhibitor azidothymidine (AZT), and the protease inhibitor nelfinavir (NFV), to inhibit TF and CC CH058. Strikingly, the TF was relatively resistant to both AZT and NFV (Fig 6A–6D), reminiscent of the resistance to IFN exhibited in Figs 1 and 5. Given the absence of sequence diversity in the protease and RT of the TF and CC viruses, these data strongly suggest that enhanced replicative fitness underlies the apparent resistance of this transmitted HIV-1 to antiretroviral compounds. To further investigate this observation, we used another matched pair of viruses (CH040) and considered the sensitivity of the CH040 TF and CC viruses to IFN and AZT. Crucially, although The CH040 TF and CC differ at two amino acids within Pol, RT is identical in both the TF and CC. We additionally observed a substitution during the rescue and generation of the CH040 CC virus stocks (Env V180E), not present in the TF or CC IMC (that possibly arose due to the reduced fitness of the CH040 CC (Fig 1A)). Nonetheless, similar to our observations using the CH058 pair, the TF virus was more resistant to IFN (Fig 7A and 7B) and was also more resistant to AZT (Fig 7C and 7D). These data are consistent with the idea that apparent resistance to IFN and antiviral compounds can be mechanistically linked.

The illustrations in Fig 4 indicate that when a sufficiently large growth rate impediment (mimicking the antiviral state induced by IFN) is applied to the simulated growth curves, the slower-growing virus appears undetectable, whereas the faster growing virus grows exponentially and overwhelms the culture (Fig 4A). However, most of the data presented in Figs 1D, 3E, 6A and 6C resembles scenarios in the middle of Fig 4A, with few examples of panels that resemble the binary scenarios towards the bottom right of Fig 4A (where one virus is in the lag phase when the other is in an exponential growth phase). Instead, we observe multiple examples where replication of both the TF and the CC has been suppressed so that the lag phase continues for the duration of the experiment (Fig 1D (1 pg/μl), Fig 3E (Mx2) and Fig 6C (150μM)). We speculate that this is because very small differences in the numbers of cells initially infected can substantially influence the duration of the lag phase (beyond the duration of the experiment). Nonetheless, there are examples of binary scenarios such as Fig 3E (IFITM2) and with both IFN and AZT, in which exponential propagation of the CH040 TF was observed at concentrations where infection by the CC was effectively suppressed. These observations again emphasise that small differences in replicative fitness could lead to quite stark differences in inhibitory phenotypes.

### TF viruses overwhelmingly exhibit prevalent residues at polymorphic sites

**Fig 8:**
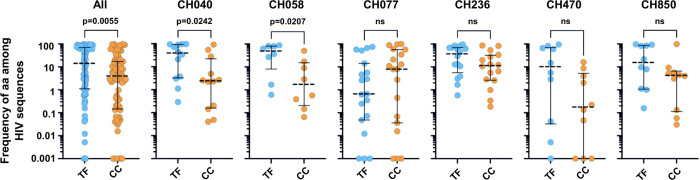
Transmitted HIV-1 tends to access more prevalent sequence space than matched chronic viruses. Amino acid substitution (or variation) frequencies for sites that exhibit amino acid divergence between the matched TF/CC pairs are shown using between 4568 sequences (Tat) and 19237 sequences (Nef) for subtype B and between 1548 sequences (Rev) and 4345 sequences (Env) for subtype C using sequences from the Los Alamos sequence database for each viral protein. Each point represents one of the amino acid sites that differs between the TF and CC in that virus pair. Medians and interquartile ranges are indicated and significance was determined using a Mann-Whitney test.

A common mechanism underlying the relative resistance of transmitted HIV-1 to IFN, protease inhibitors and RT inhibitors is a more parsimonious explanation than a requirement for multiple independent resistance mechanisms. We therefore considered whether there were sequence signatures that might correlate with our *in vitro* observations. There is a tendency for more consensus-like HIV-1 variants to be transmitted [21,32], and additional HIV-1 studies have also shown that amino acid prevalence and fitness can be closely linked [58–60] (although consensus-like variants and transmitted variants are not always fitter than their non-transmitted counterparts [21,25]). To carefully characterise any sequence changes between the TF and CC viruses investigated in this work (CH040, CH058, CH236 and CH850), we confirmed the IMC sequence of these pairs via Illumina MiSeq. The sequenced IMCs had 100% coverage with a minimum mean depth of 5229 (S4 Fig). Two additional pairs investigated in a foundational paper describing the resistance of TF viruses to IFN (CH077 and CH470) were also included in our analysis [20]. The sequence analysis allowed us to identify the amino acid sites that exhibited differences between the matched TF/CC pairs (‘divergent TF/CC sites’) and allowed us to examine the amino acid frequencies at these divergent sites in the context of a reference HIV-1 sequence, HxB2 (S5 Fig). In order to consider the frequencies of amino acids at divergent TF/CC sites in the context of a more global representation of HIV-1 sequences and of genome evolution, we next obtained all available sequences of HIV-1 subtype B and subtype C from the Los Alamos HIV sequence database (www.hiv.lanl.gov/). Strikingly, when we evaluated amino acid usage of HIV-1 at the divergent TF/CC sites for all Los Alamos sequences (of the relevant subtype), when all the pairs were considered, we found that the TF viruses tended to utilise residues that were significantly more frequently accessed by HIV-1, while the CC viruses tended to use residues that were used less frequently (p = 0.0055) (Figs 8 and S5). This difference was substantial, with the population of substitutions in the CCs visibly shifted towards lower prevalence, corresponding to an ~3-fold decrease in the median prevalence of the residues accessed by the CC viruses. While the overall trend was clear, we note that differences observed within 4 of the 6 individual pairs analysed were not significant (Fig 8). This trend of TF viruses accessing more frequently utilised sequence space at the divergent TF/CC positions seems likely to be a consequence of these residues conferring increased replicative fitness. We speculate that this trend could be due to constraints that are absent in a new host (such as acquired immune attack or antiretroviral therapy), selecting for transmitted variants that access optimal sequence space for replication in a naïve host. To this end, when we used CH058 to investigate how many sites of change were associated with immune escape using the HIV mutation browser (https://hivmut.org/) [61], we found that seven out of eight sites that differed between TF and CC viruses had publications associated with drug or immune escape (*gag* 248 [62,63], *env* 232 [64], 339 [65,66], 588 [67], 747 [68], *rev* 54 [69], and *nef* 108 [70,71]).

Interestingly, amongst the virus pairs tested, CH077 contrasts from this observed trend, as the distribution of conserved frequencies appears lower for the TF than the CC CH077 virus. CH077 is also observed to be an outlier in a recent work investigating the fitness of TF/CC pairs [29]. In that study, no significant fitness difference was detected from a single passage competitive fitness assay between the TF and the CC virus, and a difference in fitness could only be visualised by passaging the mixture of cell-free viruses three times [29].

## Discussion

The propensity of TF viruses to be IFN-resistant is well-documented, and, although not universally observed, is described as an important determinant of successful HIV-1 transmission [18,20,26]. However, the absence of recurrent sequence signatures suggesting innate immune attack in transmitted HIV-1, alongside a limited number of reports describing the relative resistance of transmitted HIV-1 to specific ISGs, means that little is known about the identity of the molecular defences that might constrain HIV-1 transmission and select for relatively resistant transmitted HIV-1 variants. We sought to fill this knowledge gap by identifying the specific molecular defences that make CC viruses more susceptible to the IFN-induced antiviral state than TF viruses. Using arrayed ISG expression screening, we identified multiple ISGs that could inhibit both our reporter TF and CC viruses. Unexpectedly, for the majority of ISGs tested (9/10), the CC virus was more sensitive to ISG-mediated inhibition than the TF virus. Moreover, because the only ISG that the TF did not appear relatively resistant to (Mx2) was also the most inhibitory ISG tested (suppressing both TF and CC viruses for the duration of the experiment), it remains possible that the TF virus is relatively resistant to all the ISGs we considered. Thus, the consistent ISG resistance exhibited by transmitted HIV-1 hinted at a single, common, underlying mechanism.

Both relative resistance to specific antiviral defences [24,28] and improved replicative fitness [26,29] have been described in other work as characteristics of transmitted HIV-1. We compared both characteristics using simple simulations, which illustrated that small differences in virus growth rate between a virus pair were amplified by logistic growth in the presence of growth rate inhibitors that mimicked IFN treatment, making the faster growing virus appear far less sensitive to the inhibitor. Our subsequent statistical modelling found no support for the hypothesis that a difference in IFN sensitivity was required to explain the relative IFN resistance of the CH058 TF. Instead, our models exclusively used a minor difference in replicative fitness to closely fit our *in vitro* data, even when an effect describing variable sensitivity was made available. Indeed, a strong correlation between HIV-1 replicative fitness and IFN resistance has been reported previously [21], and the idea that reduced replicative fitness can mechanistically underpin increased IFN sensitivity has previously been proposed as a general process that could tip the balance in favour of the host and influence virus pathogenesis and host range [72–74].

Importantly, the difference in growth rate between TF and CC CH058 suggested by our fitted logistic growth models was relatively small (17%). Previous work investigating the genetic fragility of HIV-1 capsid revealed that the majority of amino acid substitutions in capsid caused a far greater fitness reduction (70% of random single amino acid changes in capsid caused at least a 50-fold reduction in replicative fitness [60]). Additionally, research into the fitness landscape of HIV-1 Gag revealed that making multiple sequence changes that were predicted to impact fitness (based on sequence prevalence) also resulted in differences in replicative capacity much greater than the difference observed in the CH058 pair [59]. Indeed, the changes in replicative fitness that could meaningfully impact IFN sensitivity are small enough that they could be easily overlooked and can likely be mediated by substitutions in many viral proteins. Accordingly, the TF and CC pairs we analysed indicated that transmitted HIV-1 tended to utilise more frequently accessed sequence space (that could plausibly confer higher replicative fitness) than the CC viruses, in line with previous observations [21,32].

One prediction from the hypothesis that replicative fitness underpins IFN resistance is that other inhibitors of viral growth should be similarly resisted. To examine this, we designed an experiment centred around sensitivity to inhibitors that target enzymes (protease and reverse transcriptase) that have identical sequences in the matched CH058 and CH040 TF/CC pairs. The striking observation that both the CH058 and CH040 TFs were more resistant to antiretroviral compounds than their matched CC counterparts (Figs 6–7), despite having identical target protein sequences, is consistent with an underlying role for enhanced replicative fitness in conferring resistance. Moreover, this phenomenon could possibly be broadly applicable and echoes previous work that found that increased replicative fitness, conferred by the *gag* gene, correlated with resistance to protease inhibitors [23]. Furthermore, it seems possible that other reported TF/CC phenotypes could also possibly be explained by replicative fitness [28]. In particular, Hertoghs *et al*. showed that when other matched HIV-1 TF/CC pairs were studied in Langerhans cells (LCs), most of the TF viruses tested were able to infect LCs, whereas matched CC viruses had lost this ability [28]. Similarly, the reported resistance of TF viruses to inhibition by IFITMs [24] could also potentially be explained by enhanced replicative fitness. Importantly, this common resistance mechanism does not diminish the pivotal role these factors could play in constraining transmission. Additionally, research into hepatitis C virus (HCV) has also shown that high replicative fitness is linked to increased resistance to antiviral agents [75,76] and resistance to lethal mutagenesis [77]. Moreover, as noted previously [23], there is a tendency for virologists to think of resistance to antiviral compounds and immune defences as a trade-off, where decreased replicative fitness is a cost of specific resistance mutations. While this is an important and fundamental concept, the role of replicative fitness in overcoming immune defences and therapeutic interventions requires more research.

Importantly, we did not design this study as a specific investigation into the role of replicative fitness in defining the IFN resistance of HIV-1. Thus, this study should be considered as exploratory and how generalisable the conclusions are warrants further investigation. However, there are reasons to believe that it is possible that replicative fitness could underpin the majority of cases where transmitted HIV-1 is observed to be comparatively IFN resistant. Notably, where IFN resistance is reported, there is often evidence that the same IMCs/isolates possess higher replicative fitness. For example, the TFs from CH058, CH077 and CH470 (subtype B) and CH236 and CH850 (subtype C) are known to be relatively IFN resistant [20,21]. Crucially, multiple groups have demonstrated that these same TFs have more replicative capacity than their matched controls (in primary cells) using distinct methodologies [21,29]. Similarly, a study comparing 300 subtype B and C isolates from eight transmission pairs demonstrated that recipient isolates possessed higher replicative fitness as well as being more IFN-resistant [26]. Our simple, illustrative, logistic growth simulations (Fig 4) suggest that the 1.2–1.7-fold increase in replicative capacity reported by Iyer *et al*., could plausibly underpin the reported IFN resistance observed in this study [26] (although further work is required to ascertain if this is the case). In addition, a comparison of 6 subtype C TFs and 12 non-transmitted counterparts demonstrated a strong correlation between replicative fitness and IFN resistance [21]. Considered together, these studies and the observations reported herein suggest it is possible that IFN resistance could be a frequent and likely consequence of enhanced replicative fitness. In addition, if IFN resistance is underpinned by enhanced replicative fitness, this could potentially explain the apparent lack of sequence signatures in transmitted HIV-1 indicative of the evasion of a specific antiviral effector (or effectors).

Our study is consistent with the idea that acquired immune responses increasingly drive chronic HIV-1 into a constrained sequence space that is resistant to immune attack but has less replicative capacity (when considered in the absence of immune attack). During transmission to a naïve host, the immune-resistant variants (possessing lower replicative fitness when examined in the absence of immune attack) are outcompeted by their fitter, immune-sensitive, more consensus-like counterparts (that perhaps originate from a reservoir established earlier in infection). It is important to note that this scenario is not consistent with all the available data, as in some cohorts transmitted HIV-1 from subtype B [25] and subtype C [21] did not tend to possess more replicative capacity or increased IFN resistance. It is therefore currently unclear why transmitted HIV-1 is IFN resistant in some studies, while in other studies it is not. One potential explanation is that despite the formidable amount of work that has been done in this area, a relatively small number of transmission pairs have been analysed [21,25,26]. Because so many variables such as HIV-1 subtype, the stage of infection/viral load of the donor [11,31], mechanical damage, route of exposure, coinfection/inflammatory status [15], host genotype and gender all influence HIV-1 transmission, each transmission event is somewhat unique. Thus, a large number of transmission events may need to be analysed before the relative contribution of phenotypic properties that favour transmission in some, but not all, instances can be understood. For example, large differences in the IFN sensitivity of HIV-1 isolates at different stages of infection [31] could influence the relative replicative fitness/IFN-resistance of a transmitted variant (compared to non-transmitted counterparts) derived from donors at different stages of infection. In cases where IFN resistance reflects enhanced replicative capacity, it will be difficult to determine what the underlying selection pressure for these correlates could be (i.e., whether IFN-resistance, replicative capacity or something else selected for at the point of transmission).

In this study, we used illustrative simulations and standard logistic growth-based regression models to guide the interpretation of conventional *in vitro* experiments and link existing observations in the field [20,21,23,26, 29]. Focusing on two pairs of viruses, we demonstrate that the observable relative IFN resistance of transmitted HIV-1 could possibly be achieved through enhanced replicative fitness, as opposed to resistance to specific antiviral effectors (although further work is required to ascertain how generalisable this IFN resistance mechanism might be). Indeed, we speculate that it is possible that relative replicative fitness and relative IFN resistance are generally mechanistically linked properties of viruses (again, many further studies would be required to further support this possibility). Notably, a nonspecific mechanism does not downplay the potential importance of IFN resistance as a key phenotypic property of transmitted HIV-1 (or any other virus). Moreover, nonspecific IFN resistance in no way depreciates the pivotal role that IFN responses likely play as a barrier to HIV-1 transmission [5], although much work is still required to understand how strong and variable this barrier might be to natural transmission events.

## Materials and methods

### Cells

Adherent HEK 293T cells were propagated from lab stocks maintained in Dulbecco’s modified Eagle’s medium (DMEM) supplemented with 10% fetal calf serum (FCS) and 10 μg/ml gentamicin. Suspension MT4 cells were expanded from lab stocks and maintained in RPMI medium supplemented with 10% FCS and 10 μg/ml gentamicin. MT4-LTR-GFP indicator cells (TMZR5 cells) have been modified to express the CCR5 receptor and contain a cassette in which hrGFP expression is driven by the HIV-1 LTR and have been described previously [2,39]. The MT4 CCR5-R126N cells, referred to as MT4-R5 cells in this work, were produced through the PCR of genomic DNA extracted from TMZR5 cells to generate the R126N CCR5 product, and to also introduce SfiI restriction sites at the 5’ and 3’ ends of CCR5 gene, enabling cloning into an MLV-based vector (primer pair AA-099-LPCX CCR5-F 5’-CTCTCTGGCCGAGAGGGCCATGGATTATCAAG TGTCAAGTCCAATC-3’ and AA-100-LPCX CCR5-RC 5’-TCTCTCGGCCAGAGAGGCCTCACAAGCCCACA GATATTTCCTGC-3’). Following transduction of MT4 cells, a limited dilution strategy was implemented to select a cell line that fostered replication and maintained IFN sensitivity. All lentivirus transduced cells were selected and cultured in medium supplemented with 2 μg/ml puromycin (Melford Laboratories), 5 μg/ml blasticidin (Melford Laboratories) or 1 mg/ml G418 (Invitrogen), as appropriate.

### Retroviral vectors and plasmids

The SCRPSY (KT368137.1) lentiviral vector has been previously described [2], pLV-EF1a-IRES-Neo (Addgene plasmid #85139) was modified to include SfiI sites flanking the transgene ORF by inserting the TagRFP (or gene of interest) ORF with flanking SfiI sites between the unique BamHI and EcoRI restriction sites using PCR (primer pair AW177-BamHI-SfiI-RFP-F’ 5’-CTCTCGGATCCGGCCGAGAGGGCCATGAGCGAGCTGATTAAG-3’ and AW178-EcoRI-SfiI-RFP-R’ 5’-CTCTCGAATTCGGCCAGAGAGGCCTCACTTGTGCCCCAG-3’). Gene editing was achieved using the lentiCRISPRv2-Blast system [78].

### Replication competent viruses

HIV-1 stocks (Table 1) were generated through transient transfection of HEK 293T cells in the presence/absence of pCMV-VSV-G using polyethylenimine (PEI). The following clones were used: replication-competent GFP-encoding pNHG (JQ585717) [51,79], and a panel of full-length transmitted/founder (TF) and matched chronic control (CC) HIV-1 infectious molecular clones that were obtained as generous gifts from Beatrice Hahn and Stuart Neil. In all cases, supernatants were harvested at ∼48 h post transfection and clarified using a 0.45-μm-pore-size filter and stored at -80°C. CH058 and CH040 working stocks were additionally propagated for 10 days in TMZR5 cells after transfection. Virus stocks were validated by deep sequencing, and we note that 2 predominant changes (relative to the IMC plasmid) were detected in the CH040 CC stocks used in Fig 7. One is a synonymous change T1875C (V362V in Gag), and one is a nonsynonymous change T6759A (V180E in Env). The CH040 TF has a V at position 180 in Env. These occurred during rescue/propagation and were not present in the IMC plasmid.

**Table 1:** Viruses used in this study.

Virus (subtype) and designation	Description	Accession number	Co-receptor usage	Reference(s)
**HIV-1 Group M (B)**				
NHG	GFP in place of *nef*	JQ585717	X4	[51]
CH058-GIN	TF	MW535546	R5	This Study
	CC GFP and IRES inserted between *env* and *nef* genes	MW535544	R5	
CH040	TF	MW535542	R5	[17]
	CC (Env V180E in the stocks used for Fig 7)	MW535541	R5	
CH058	TF	MW535545	R5	[17]
	CC	MW535543	R5	
**HIV-1 Group M (C)**				
CH236	TF	MW535549	R5	[20]
	CC	MW535548	R5	
CH850	TF	MW535552	R5	[20]
	CC	MW535551	R5	

### IFNα14 production and quantification

Stat1 deficient U3A fibroblasts, a generous gift from Stephen Goodbourn, were utilised to minimise the presence of secreted ISGs in IFN preparations. These U3A cells, which lack STAT1, were modified to produce IFN under a doxycycline-inducible system. In order to efficiently generate high quantities of IFNα14, engineered U3A cells expressing IFNα14 were seeded into 10-cm dishes at a ratio of 1:3 to achieve maximum confluency prior to stimulation with 125 ng/ml of doxycycline (DOX). The DOX treated cells were incubated for 24 hours to allow sufficient expression of IFNα14 before the supernatants were harvested and purified using a 0.45-μm filter. The biological units of recombinant human IFNα14 produced in this study were determined using ISRE-GFP expressing HEK293T cells. Cell-free supernatants containing IFNα14 were 1.5-fold serially diluted and titrated onto 2.0 x 10^5^ cells/well of ISRE-GFP cells in a 96-well plate. Titration of IFNα14 was carried out in parallel with commercial IFN, where commercial IFN stocks were used to generate a standard curve for a dose determination. Based on the calculation, the estimated concentration of IFNα14 was 1153.2 pg/μl. To assess the toxicity of IFNα14 treatment, the LIVE/DEAD fixable green dead cell stain kit (Invitrogen) was used.

### Arrayed ISG expression screening

The ISG overexpression screening was completed similarly to previously described protocols [2,46]. In short, MT4-R5 cells were seeded in 96-well plates and transduced with a library of ISG-encoding SCRPSY vectors (one ISG per well) containing 527 unique human ISG open reading frames. 48 hours after transduction, cells were split into two new plates and infected with a GFP-encoding virus. For single-cycle infections with NHG, 100 μg/ml dextran sulphate was added to the cells at 16 hours post-infection to limit viral spread, and cells were fixed at 48 hours post-infection in 4% formaldehyde. For multi-cycle infections using the CH058 GIN viruses, cells were fixed at 96 hours post-infection in 4% formaldehyde. Fixed cells were analysed with flow cytometry using a Guava EasyCyte system (Luminex). Subsequent validation screens of ISG ‘hits’ and candidate effectors were conducted with independent lentiviral preps using the same methods. For the exclusions screens, MT4 and TMZR5 cells were transduced with the genes in Fig 2G as before and 96 hours post-transduction the supernatants were harvested to measure toxicity of the expressed ISGs using the CytoTox-Glow kit (Promega). In a separate experiment, MT4 cells were transduced with the same genes and 96 hours post-transduction the cells were fixed in 4% formaldehyde and stained using the LIVE/DEAD Fixable Red Dead Cell Stain Kit (Invitrogen) to assess the viability of the transduced cells. In a similar fashion MT4-ISRE-GFP cells were transduced and at 96 hours post-transduction cells were fixed in 4% formaldehyde to measure ISRE induction (GFP-positive cells) as surrogate for IFN induction.

### Virus infections and titrations

Suspension cells were seeded immediately prior to infection or treatment. For experiments involving IFN treatment, IFNα14 produced as described above was added 24 hours prior to infection. Azidothymidine (3485) and Nelfinavir (4621) were obtained from the NIH AIDS Reagents Program (catalogue numbers indicated in parentheses) and added 2 hours prior to infection using the indicated concentrations. Virus titrations were carried out as previously described [27]. Cells were infected with a titrated challenge of serially diluted virus-containing supernatant. Cell lines were treated with polyanionic dextran sulfate 17–18 hours post-infection to limit infection to a single cycle (where single cycle infection is indicated). At 48 h after virus challenge, levels of infection were determined via flow cytometry, for either GFP-encoding viruses or GFP-reporter TMZR5 infected cells. The titres plotted are the mean of triplicate (n = 3) estimations of the titre extrapolated from different doses within the linear range (error bars represent the standard deviation). For spreading assays, cells were infected with a dose of HIV-1 that resulted in 0.01–0.1% of cells GFP+ 24 h post infection. The virus inoculum required for this experiment was calculated based on the number of single-cycle infectious units determined in TMZR5 indicator cells. Cells were sampled every 24 h, fixed, and the levels of infection determined by flow cytometry. To maintain relatively constant cell growth and prevent overgrowth of the culture, cells were split each day from 48 h post infection by replacing a fixed amount (1/10^th^– 1/5^th^, depending on the experiment) of the culture with fresh medium. Experiments were conducted in quadruplicate (n = 4). A typical result from at least two independent experiments is shown. For the AUC analysis, where cultures were overwhelmed (available target cells exhausted), indicated by inflection in the curve indicating the level of infection (at high percentages), the peak recorded percentage in that culture was used for the remaining timepoints in the AUC analysis. This method avoids the artificial AUC inflation of inhibited/attenuated viruses (that take far longer to overwhelm the culture) and crucially still underestimates the phenotypic differences reported here (as longer exponential phases would increase the differences in the numbers of infected cells caused by different underlying growth rates). Similarly, points after the cultures have become overwhelmed have not been plotted/sampled in Figs 1D, 3E, 6A and 6C as in these instances viral propagation has been limited by the culture conditions as opposed to the properties of the virus.

### Infectious yield assays

TMZR5 cells were seeded in 6-well plates and treated with increasing doses of IFNα14. 24 hours after IFN treatment, cells were challenged with HIV-1 at an MOI of 0.5. At 6 hours post-infection, cells were washed once with PBS and pelleted by centrifugation. Supernatant containing inoculum was removed and fresh medium containing the appropriate dose of IFN was used to resuspend the cell pellet and transferred into a fresh 6-well plate. At 46 to 48 hpi, supernatant containing virus was harvested and filtered using a 0.45 μm filter and the infectivity of the virus was estimated via titration.

### Western blot analyses

For preparation of cell lysates, cell pellets were resuspended in protein sample buffer (12.5% glycerol, 175 mM Tris-HCl [pH 8.5], 2.5% SDS, 70 mM 2-mercaptoethanol, 0.5% bromophenol blue). Proteins were subsequently separated on NuPage 4% to 12% Bis-Tris polyacrylamide gels and transferred onto nitrocellulose membranes. Blots were probed with either anti-actin (JLA20 hybridoma; courtesy of the Developmental Studies Hybridoma Bank, University of Iowa), anti-CD38, anti-CD80, anti FNDC3B, anti-SCARB2 (25284-1-AP, 14292-1-AP, 22605-1-AP, 27102-1-AP; Proteintech) anti-MICB (VPA00747; Bio-Rad) or anti-TMEM140 (SAB1304546; Sigma) primary antibodies. Thereafter, membranes were probed with fluorescently labelled goat anti-rabbit or goat anti-mouse secondary antibodies (Thermo Scientific) and scanned using a LiCor Odyssey scanner.

### Illustrative logistic simulations (in which one parameter is varied)

To illustrate the effect of small differences in growth rate on growth curves generated in the presence of a growth-inhibiting substance, we simulated a logistic growth process for two viruses:

$$x_{v,t}=\frac{kx_{v,0}\exp\left(r_{v}^{\prime }t\right)}{k+x_{v,0}[\exp\left(r_{v}^{\prime }t\right)-1]}$$

where *x*_*v*,*t*_ is the number of cells infected by virus *v* at time *t*, *x*_*v*,*0*_ is the initial number of infected cells (here fixed to 100), and *k* is the carrying capacity (fixed to 10 000 in all simulations). Finally, *r′*_*v*_ is the effective or realized growth rate of virus *v*, calculated as described below. Viruses were assumed to be growing independently (i.e., in separate wells). To allow different growth rates, the growth rate of virus two was scaled relative to that of virus one by a factor *s*:

$$r_{2}=r_{1}s$$

In all of these illustrative simulations, r_1_ was held constant at 3, broadly similar to the growth rate measured for CH058 TF (Fig 5D). Similarly, the level of growth rate inhibition, *i*, was allowed to vary between viruses by a scaling factor *c*:

$$r_{1}^{\prime }=\max\left(0,r_{1}-i\right)$$

$$r_{2}^{\prime }=\max\left(0,r_{2}-ci\right)$$

$$=\max\left(0,r_{1}s-ci\right)$$

In the first set of illustrative simulations, viruses differed in growth rate only, with the second virus having a lower growth rate than the first. This was achieved by varying *s* from 0.6 to 0.95 (i.e., virus two’s growth rate was scaled to between 60% and 95% of virus one’s growth rate) while the inhibition scaling factor *c* was fixed at 1, giving both viruses equal sensitivity to the growth rate inhibitor. In the second set of illustrative simulations, the underlying growth rates of both viruses were equal (*s* = 1), but virus 2 was more sensitive to the growth rate inhibitor (*c* > 1). In these illustrative simulations, the scaling factor *c* was varied from 1.1 (virus two is 10% more sensitive than virus one) to 1.8 (virus two is 80% more sensitive). In both sets of illustrative simulations, the level of inhibition, *i*, was varied such that *r*_*1*_ would be reduced by between 0 and 90% (Fig 4).

### Logistic growth-based regression models

To test whether the observed differences in growth curves were the result of growth rate differences, differences in sensitivity to IFN, or both, the *in vitro* propagation assays above were repeated over a targeted range of IFN concentrations. A maximal dose of 0.5 pg/μL was chosen, as in the initial IFN spreading assays this dose enabled a clear difference between the TF/CC pair with minimal IFN-associated toxicity (~80% live cells). The remainder of doses were spread at 0.1 pg/μL intervals to capture incremental differences in growth rate.

Data from these assays were modelled as a logistic growth process:

$$x_{t,v,j,d}=\frac{k_{j,d}x_{1,v,j,d}\exp\left(r_{v,d}^{\prime }t\right)}{k_{j,d}+x_{1,v,j,d}[\exp\left(r_{v,d}^{\prime }t\right)-1]}$$

Where *x*_*t*,*v*,*j*,*d*_ is the number cells infected at time *t* by virus *v*, in replicate *j* of a given treatment with IFN dose *d*, and *x*_1,*v*,*j*,*d*_ is the initial number of infected cells in this replicate (as measured at the first timepoint, 24 hours post inoculation). To account for IFN-toxicity to cells at higher doses, the maximum number of cells available to be infected (i.e., the carrying capacity, *k*_*j*_) was modelled as a function of IFN dose (*d*):

$$k_{j,d}=\beta_{k,0}+\beta_{k,1}d+u_{j,d}$$

where *β*_*k*,0_ is the mean carrying capacity when no IFN is present, *β*_*k*,1_ is the effect of 1 pg/μl IFN, and *u*_*j*,*d*_ is a random effect allowing variation in the number of cells available between different replicates of a given treatment.

In the most complex model fitted (here termed the differential sensitivity model), the achieved growth rate of each virus, *r′*_*v*,*d*_, was modelled as a function of IFN dose, a virus-specific adjustment allowing growth rates to vary between viruses, and an additional virus-specific adjustment for interferon-sensitivity:

$$r_{v,d}^{\prime }=\beta_{r,0}+\beta_{r,1}v+\beta_{r,2}d+\beta_{r,3}dv$$

Here, *v* = 0 for the TF virus and 1 for the CC virus. As a result, *β*_*r*,0_ is the growth rate of the TF virus in the absence of IFN (here termed the baseline growth rate), while *β*_*r*,1_ is the adjustment needed to achieve the baseline growth rate of the CC virus. Finally, *β*_*r*,2_ measures the baseline effect of 1 pg/μl IFN on the growth rates of both viruses, while *β*_*r*,3_ allows the CC virus to be more or less sensitive to a given IFN dose than the TF virus. The fit of this model was compared to one without the additional virus-specific adjustment for interferon-sensitivity (i.e., without the *β*_*r*,3_*dv* term), here named the constant sensitivity model.

Models were fit by maximum likelihood using version 3.1–149 of the nlme library in R version 4.0.2 [80,81]. Confidence intervals for all parameter estimates were generated by re-fitting models to 1000 hierarchical bootstrap samples of the data. For each IFN dose, the available data were truncated as soon as growth curves declined by more than 30% relative to the previous timepoint, with models fitted to the remaining data only. This was needed to accommodate the long timescale of these experiments, where both the accumulation of dead cells due to virus infection and release, and the toxicity effects of long-term culture in the presence of IFN, results in a reduction in viable cells that can be infected (Fig 5B). The sensitivity of models to this exclusion was assessed by evaluating a range of cut-off points (including no data removal). Truncation affected primarily the estimated carrying capacity and associated effect sizes (*β*_*k*,0_ and *β*_*k*,1_), with carrying capacity under-estimated when the declining parts of growth curves were included. All other parameter estimates remained broadly similar with overlapping confidence intervals, regardless of the cut-off used, and the differential sensitivity model remained unsupported.

### HIV-1 plasmid sequencing and assembly

40 ng of each plasmid DNA was sheared into approximately 350 base pair in length by sonication using a Covaris Sonicator LE220 (Covaris). Fragmented DNA was uniquely index tagged with NEBNext Multiplex Oligos for Illumina (New England Bio-Labs, E7780S and ES7600S). The Kapa LTP Library Preparation Kit (KAPA Biosystems, Roche7961880001) was deployed in this process. Libraries were quantified and quality controlled with Qubit dsDNA HS kit (ThermoFisher) and Agilent 4200 Tapestation System (Agilent). Equimolar amounts of each library were pooled together and sequenced on the Illumina MiSeq platform using MiSeq Reagent Micro Kit v2 (2x 150-cycles). Plasmid sequences were assembled using SPAdes v3.10.1 with multiple k-mer sizes. Minimum depth of 100 reads and Phred quality of 30 were used for consensus calling of the assembled sequences.

### Analysis of HIV-1 sequences

Using a procedure outlined in [27] to determine the frequency of each amino acid, the Los Alamos National Database (http://www.hiv.lanl.gov/) was used to download all gene sequences available ranging from 4568 sequences for tat to 19237 for nef in subtype B and from 1548 sequence in rev to 4345 in env for subtype C. Only one sequence was selected per patient. Following a codon alignment of each gene, the frequency of amino acids was determined for sites that are different between the paired TF and CC sequences.

## Supporting information

S1 Fig Because of the proapoptotic effect of IFNs, the viability of IFN-treated TMZR5 cells was also assessed in parallel cultures to those used in Fig 1. Viability was tested using flow cytometry using the LIVE/DEAD fixable dead cell stain kit (Invitrogen).
(PDF)

S2 Fig Western blot gels assessing protein expression levels and IFN induction of expression of (A) CD80, (B) FNDC3B, (C) MICB (D) TMEM140, (E) CD38, (F) SCARB2. (G) TMZR5 cells (modified to express CD38 and SCARB2) were challenged with NHG and sampled daily to monitor virus spread. GFP-positive cells were enumerated via flow cytometry. (H) Western blots of the seven CRISPR guides and non-targeting control guide cell lines for CD38 in PM1 cells. (I) PM1 cell lines were pre-treated for 24 hours with the IFNα14 doses indicated and were subsequently challenged with NHG and sampled daily to monitor virus spread. (J) Western blots of the seven CRISPR guides and non-targeting control guide cell lines for SCARB2 in TMZR5 cells. (K) TMZR5 cell lines were pre-treated for 24 hours with the IFNα14 doses indicated and were subsequently challenged with NHG and sampled daily to monitor virus spread. Viral spreading replication experiments took place on two occasions, a typical result is shown. The white line in A indicates that the CD80 image was flipped for this blot to correct sample order, but the blot is the same for both portions of this image. Raw western blot images can be viewed in S7 Fig.
(PDF)

S3 Fig Small growth rate differences become discernible under growth rate inhibition because the lag phase becomes observable. (A) Illustrative logistic growth simulation of two viruses, where the growth rate of virus two (orange) is scaled to 0.8 times that of virus one (blue). Both viruses experience the same amount of inhibition; labels above each plot indicate percent inhibition relative to the growth rate of virus 1. (B) Observed growth dynamics with and without interferon. Coloured points show means (+/- standard error) across 4 experimental replicates, while grey points show individual observations (data as in Fig 5; blue: CH058 TF, orange: CH058 CC).
(PDF)

S4 Fig Sequencing coverage statistics. (PDF)

S5 Fig Amino acid frequencies at sites that exhibit amino acid changes between sequenced TF/CC pairs are shown and compared to HxB2 (reference sequence). (PDF)

S6 Fig Raw western blot images. (PDF)

S7 Fig Raw western blot images. (PDF)
