# Supplementary material for: The apparent interferon resistance of transmitted HIV-1 is possibly a consequence of enhanced replicative fitness
Source: PLoS Pathog. 2022 Nov 18;18(11):e1010973. doi: 10.1371/journal.ppat.1010973 (PMC9718408; doi:10.1371/journal.ppat.1010973)
Supplement: S1 Fig — Viability was tested using flow cytometry using the LIVE/DEAD fixable dead cell stain kit (Invitrogen). (PDF) [file ppat.1010973.s001.pdf]

## Viability of IFN-treated TMZR5 cells

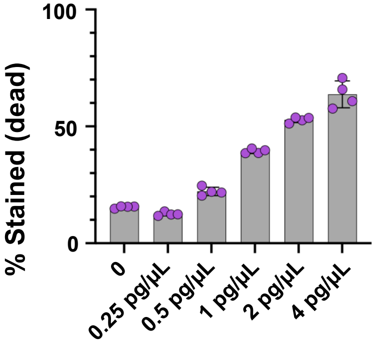


**S1.** Because of the proapoptotic effect of IFNs, the viability of IFN-treated TMZR5 cells was also assessed in parallel cultures to those used in Fig 1. Viability was tested using flow cytometry using the LIVE/DEAD fixable dead cell stain kit (Invitrogen)
