# Supplementary material for: The apparent interferon resistance of transmitted HIV-1 is possibly a consequence of enhanced replicative fitness
Source: PLoS Pathog. 2022 Nov 18;18(11):e1010973. doi: 10.1371/journal.ppat.1010973 (PMC9718408; doi:10.1371/journal.ppat.1010973)
Supplement: S2 Fig — Western blot gels assessing protein expression levels and IFN induction of expression of (A) CD80, (B) FNDC3B, (C) MICB (D) TMEM140, (E) CD38, (F) SCARB2. (G) TMZR5 cells (modified to express CD38 and SCARB2) were challenged with NHG and sampled daily to monitor virus spread. GFP-positive cells were enumerated via flow cytometry. (H) Western blots of the seven CRISPR guides and non-targeting control guide cell lines for CD38 in PM1 cells. (I) PM1 cell lines were pre-treated for 24 hours with the IFNα14 doses indicated and were subsequently challenged with NHG and sampled daily to monitor virus spread. (J) Western blots of the seven CRISPR guides and non-targeting control guide cell lines for SCARB2 in TMZR5 cells. (K) TMZR5 cell lines were pre-treated for 24 hours with the IFNα14 doses indicated and were subsequently challenged with NHG and sampled daily to monitor virus spread. Viral spreading replication experiments took place on two occasions, a typical result is shown. The white line in A indicates that the CD80 image was flipped for this blot to correct sample order, but the blot is the same for both portions of this image. Raw western blot images can be viewed in S7 Fig. (PDF) [file ppat.1010973.s002.pdf]

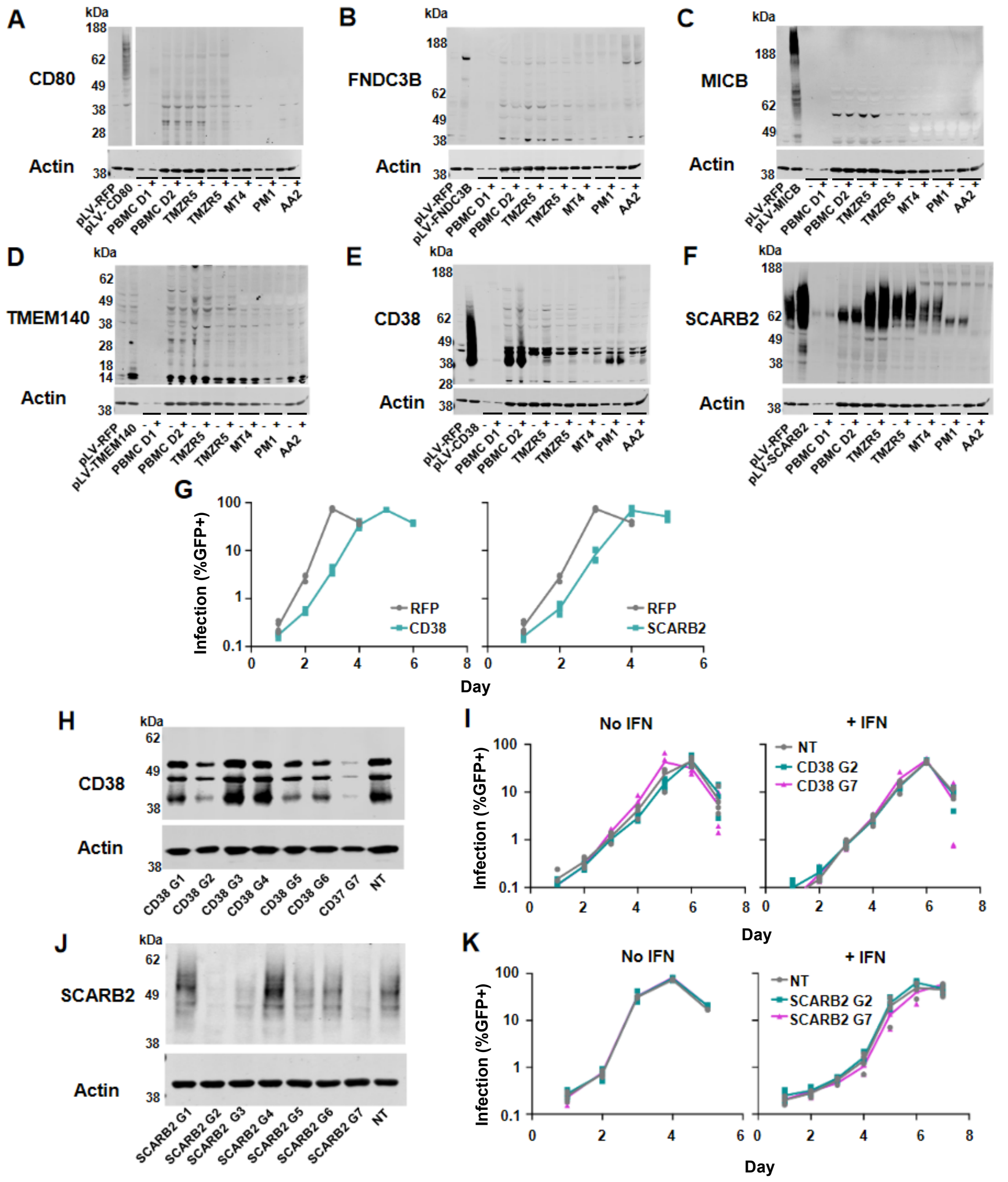

S2. Western blot gels assessing protein expression levels and IFN induction of expression of (A) CD80, (B) FNDC3B, (C) MICB (D) TMEM140, (E) CD38, (F) SCARB2. (G) TMZR5 cells (modified to express CD38 and SCARB2) were challenged with NHG and sampled daily to monitor virus spread. GFP-positive cells were enumerated via flow cytometry. (H) Western blots of the seven CRISPR guides and non-targeting control guide cell lines for CD38 in PM1 cells (I) PM1 cell lines were pre-treated for 24 hours with the IFNα14 doses indicated and were subsequently challenged with NHG and sampled daily to monitor virus spread. (J) Western blots of the seven CRISPR guides and non-targeting control guide cell lines for SCARB2 in TMZR5 cells. (K) TMZR5 cell lines were pre-treated for 24 hours with the IFNα14 doses indicated and were subsequently challenged with NHG and sampled daily to monitor virus spread. Viral spreading replication experiments took place on two occasions, a typical result is shown. The white line in A indicates that the CD80 image was flipped for this blot to correct sample order, but the blot is the same for both portions of this image. Raw western blots images can be viewed in S7.
