# Supplementary material for: The apparent interferon resistance of transmitted HIV-1 is possibly a consequence of enhanced replicative fitness
Source: PLoS Pathog. 2022 Nov 18;18(11):e1010973. doi: 10.1371/journal.ppat.1010973 (PMC9718408; doi:10.1371/journal.ppat.1010973)
Supplement: S3 Fig — (A) Illustrative logistic growth simulation of two viruses, where the growth rate of virus two (orange) is scaled to 0.8 times that of virus one (blue). Both viruses experience the same amount of inhibition; labels above each plot indicate percent inhibition relative to the growth rate of virus 1. (B) Observed growth dynamics with and without interferon. Coloured points show means (+/- standard error) across 4 experimental replicates, while grey points show individual observations (data as in Fig 5; blue: CH058 TF, orange: CH058 CC). (PDF) [file ppat.1010973.s003.pdf]

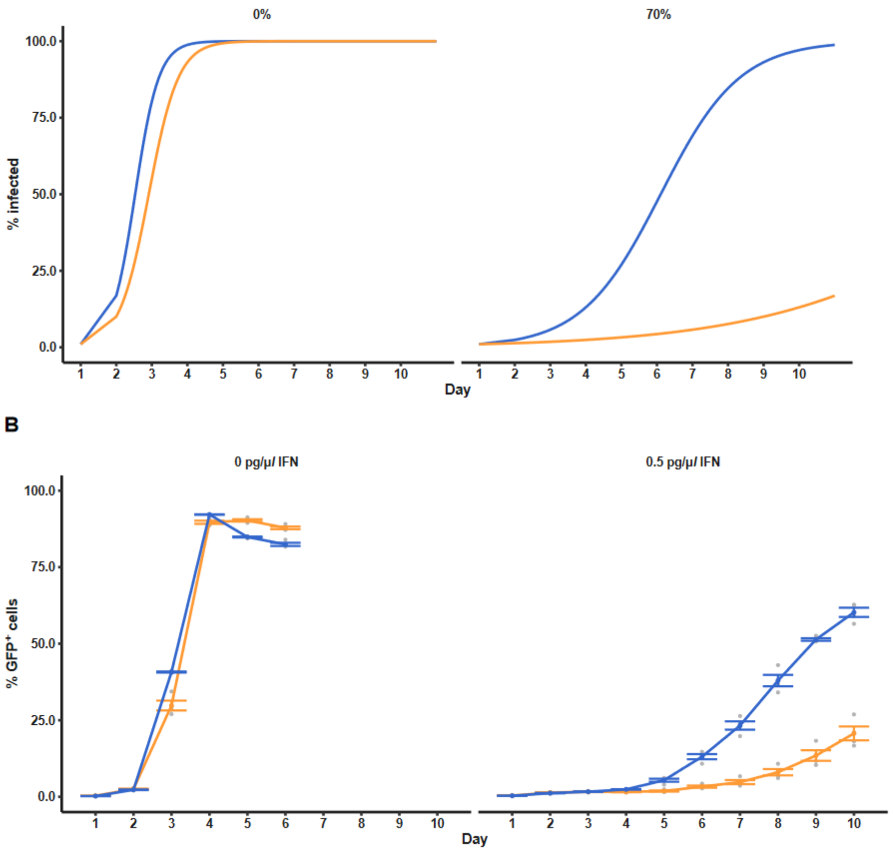

S3. Small growth rate differences become discernible under growth rate inhibition because the lag phase becomes observable. (A) Logistic growth simulation of two viruses, where the growth rate of virus two (orange) is scaled to 0.8 times that of virus one (blue). Both viruses experience the same amount of inhibition; labels above each plot indicate percent inhibition relative to the growth rate of virus 1. (B) Observed growth dynamics with and without interferon. Coloured points show means (+/- standard error) across 4 experimental replicates, while grey points show individual observations (data as in figure 5; blue: CH085 TF, orange: CH085 CC).
