# Supplementary material for: The apparent interferon resistance of transmitted HIV-1 is possibly a consequence of enhanced replicative fitness
Source: PLoS Pathog. 2022 Nov 18;18(11):e1010973. doi: 10.1371/journal.ppat.1010973 (PMC9718408; doi:10.1371/journal.ppat.1010973)
Supplement: S4 Fig — (PDF) [file ppat.1010973.s004.pdf]

S4. Sequencing coverage statistics

Sample	Start Position	End Pos	Number of reads	Coverage bases	Coverage	Mean Depth	Mean base q	Mean map q
CH040 TF	1	13088	520076	13088	100	5933.68	37.4	56.9
CH040 CC	1	13088	552845	13088	100	6302.83	37.5	55.8
CH058 TF	1	13124	589985	13124	100	6701	37.5	56.3
CH058 CC	1	13124	530845	13124	100	6045.61	37.6	57.7
CH077 TF	1	13163	496056	13163	100	5624.54	37.3	55.6
CH236 TF	1	13785	573058	13785	100	6198.24	37.3	55.9
CH236 CC	1	13785	492204	13785	100	5321.6	37.3	55.9
CH470 TF	1	14187	673479	14187	100	7083.32	37.5	55.7
CH850 TF	1	13703	653993	13703	100	7119.34	37.5	55.9
CH850 CC	1	13706	699348	13706	100	7614.87	37.4	55.8
CH058 TF GIN	1	14438	628697	14438	100	6481.14	37.5	56.5
CH058 CC GIN	1	14438	506749	14438	100	5228.8	37.4	56
