# Supplementary material for: The apparent interferon resistance of transmitted HIV-1 is possibly a consequence of enhanced replicative fitness
Source: PLoS Pathog. 2022 Nov 18;18(11):e1010973. doi: 10.1371/journal.ppat.1010973 (PMC9718408; doi:10.1371/journal.ppat.1010973)
Supplement: S5 Fig — (PDF) [file ppat.1010973.s005.pdf]

S5. Amino acid frequencies at sites that exhibit amino acid changes between sequenced TF/CC pairs are shown and compared to HxB2 (reference sequence)

Gene	Gene Nucleotide Position	Codon Position	HXB2 Codon†	HXB2 AA†	AA position	HXB2 frequency†,‡	TF codon	TF AA	TF frequency‡	Chronic codon	Chronic AA	Chronic frequency‡	Mutation
**CH040 (Subtype B)**													
gag	359	2	gct	A	120	0.966436	gct	A	0.966436	gat	D	0.001437	A120D
gag	1208	2	aga	R	403	0.444038	aga	R	0.444038	aaa	K	0.537678	R403K
pol	227	2	aag	K	76	0.941325	aag	K	0.941325	agg	R	0.04858	K76R
pol	2178	3	gaa	E	726	0.709989	gaa	E	0.709989	gac	D	0.279075	E726D
vif	185	2	gct	A	62	0.909312	gat	D	0.03733	ggt	G	0.023128	D62G
vif	365	2	aag	K	122	0.337797	aaa	K	0.337797	aca	T	0.000406	K122T
vif	500	2	acg	T	167	0.211199	aca	T	0.211199	ata	I	0.002029	T167I
vpr	253	1	tcg	S	85	0.002915	tca	S	0.002915	aca	T	0.000486	S85T
env	440	2	atg	M	147	0.008126	gaa	E	0.011158	gga	G	0.021831	E147G
env	980	2	aga	R	327	0.954639	aga	R	0.954639	aaa	K	0.026804	R327K
nef	191	2	gag	E	64	0.909185	ggg	G	0.030618	gag	E	0.909185	G64E
nef	587	2	cga	R	196	0.898529	cga	R	0.898529	caa	Q	0.025264	R196Q
**CH058 (Subtype B)**													
gag	743	2	gga	G	248	0.628183	gga	G	0.628183	gaa	E	0.000653	G248E
env	694	1	acg	T	232	0.270588	act	T	0.270588	gct	A	0.003881	T232A
env	1015	1	aat	N	339	0.787993	aat	N	0.787993	gat	D	0.028987	N339D
env	1764	3	aag	K	588	0.466465	agg	R	0.363736	agc	S	0.001455	R588S
rev	161	2	tcg	S	54	0.490059	cgg	R	0.006118	cag	Q	0.089141	R54Q
env	2239	1	cga	R	747	0.504912	gga	G	0.016859	aga	R	0.504912	G747R
env	2515	1	gct	A	839	0.871801	gct	A	0.871801	act	T	0.0057	A839T
nef	324	3	gat	D	108	0.810885	gat	D	0.810885	gag	E	0.172324	D108E
**CH236 (Subtype C)**													
gag	366	3	aca	T	122	0.002366	gat	D	0.01656	gaa	E	0.025684	D122E
pol	46	1	ttt	F	16	0.857282	ctt	L	0.132976	ttt	F	0.857282	L16F
pol	1867	1	acc	T	623	0.214321	tct	S	0.56454	cct	P	0.064296	S623P
pol	1881	3	aca	T	627	0.948855	acg	T	0.948855	aca	T	0.948855	T627T
vif	110	2	gga	G	37	0.84669	gga	G	0.84669	gaa	E	0.009872	G37E
vif	274	1	aag	K	92	0.232288	aga	R	0.696283	gga	G	0.055168	R92G
env	481	1	atc	I	161	0.220253	atg	M	0.10725	gtg	V	0.198159	M161V
env	889	1	aca	T	297	0.618182	aca	T	0.618182	gca	A	0.028308	T297A
env	1499	2	aag	K	500	0.351899	ggg	G	0.126812	gag	E	0.277791	G500E
env	1520	2	cag	Q	507	0.033142	ggg	G	0.03061	gag	E	0.824626	G507E
env	1602	3	tca	S	534	0.970771	tca	S	0.970771	tcc	S	0.970771	S534S
env	1999	1	gca	A	667	0.04603	gac	D	0.038895	aac	N	0.336939	D667N
rev	286	1	ggg	G	96	0.847545	ggg	G	0.847545	agg	R	0.105943	G96R
env	2494	1	gta	V	832	0.295052	gta	V	0.295052	ata	I	0.129344	V832I
nef	314	2	aga	R	105	0.122637	aaa	K	0.679622	aga	R	0.122637	K105R
nef	324	3	gat	D	108	0.537815	gaa	E	0.44354	gac	D	0.537815	E108D
nef	512	2	cat	H	171	0.923319	tat	Y	0.005777	ttt	F	0.001838	Y171F
nef	568	1	gca	A	190	0.951418	gca	A	0.951418	aca	T	0.003414	A190T
**CH850 (Subtype C)**													
gag	83	2	aaa	K	28	0.078743	aaa	K	0.078743	aca	T	0.040554	K28T
gag	1015	1	cca	P	339	0.844204	cca	P	0.844204	tca	S	0.052045	P339S
gag	1120	1	gct	A	374	0.214262	tca	S	0.096316	cca	P	0.031092	S374P
vif	305	2	cta	L	102	0.969222	ctg	L	0.969222	cag	Q	0.000581	L102Q
env	55	1	acc	T	19	0.009436	ata	I	0.825086	gta	V	0.057077	I19V
vpu	215	2	cac	H	72	1	cat	H	1	cgt	R	0.000302	H72R
env	646	1	cat	H	216	0.982739	tat	Y	0.011047	cat	H	0.982739	Y216H
env	1378	1	aat	N	460	0.217261	aac	N	0.217261	gga	G	0.09298	N460G
env	1379	2	aat	N	460	0.217261	aac	N	0.217261	gga	G	0.09298	N460G
env	1380	3	aat	N	460	0.217261	aac	N	0.217261	gga	G	0.09298	N460G
env	2446	1	aat	N	816	0.044649	gat	D	0.930035	aat	N	0.044649	D816N
nef	485	2	acc	T	162	0.007878	gac	D	0.001576	gcc	A	0.001313	D162A

†HXB2 is a commonly used reference strain for consistent HIV-1 DNA and protein numbering.
‡The Los Alamos National Database (http://www.hiv.lanl.gov/) was used to download all gene sequences available ranging from 4568 sequences for tat to 19237 for nef in subtype B and from 1548 sequence in rev to 4345 in env for subtype C. Only one sequence was selected per patient. Following a codon alignment of each gene, the frequency of amino acids was determined for sites that are different between the paired TF and CC sequences for the relevant subtype.
