# Supplementary figures and images for: The apparent interferon resistance of transmitted HIV-1 is possibly a consequence of enhanced replicative fitness
Source: PLoS Pathog. 2022 Nov 18;18(11):e1010973. doi: 10.1371/journal.ppat.1010973 (PMC9718408; doi:10.1371/journal.ppat.1010973)

S6. Raw western blot images used in Fig 2

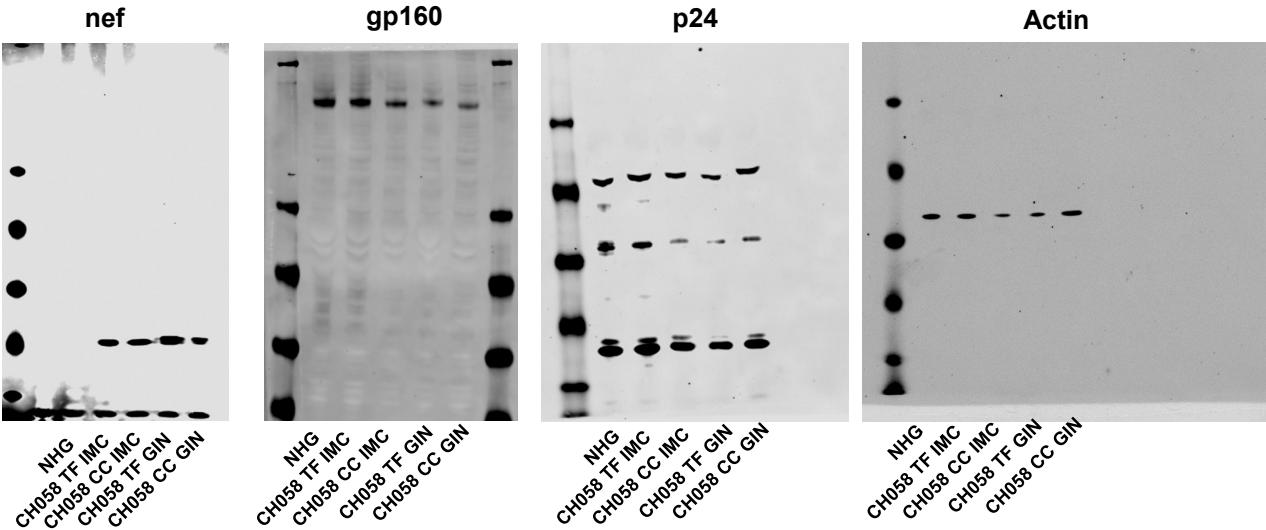

Supplement: S6 Fig — (PDF) [file ppat.1010973.s006.pdf]

S7. Raw western blot files used in S2

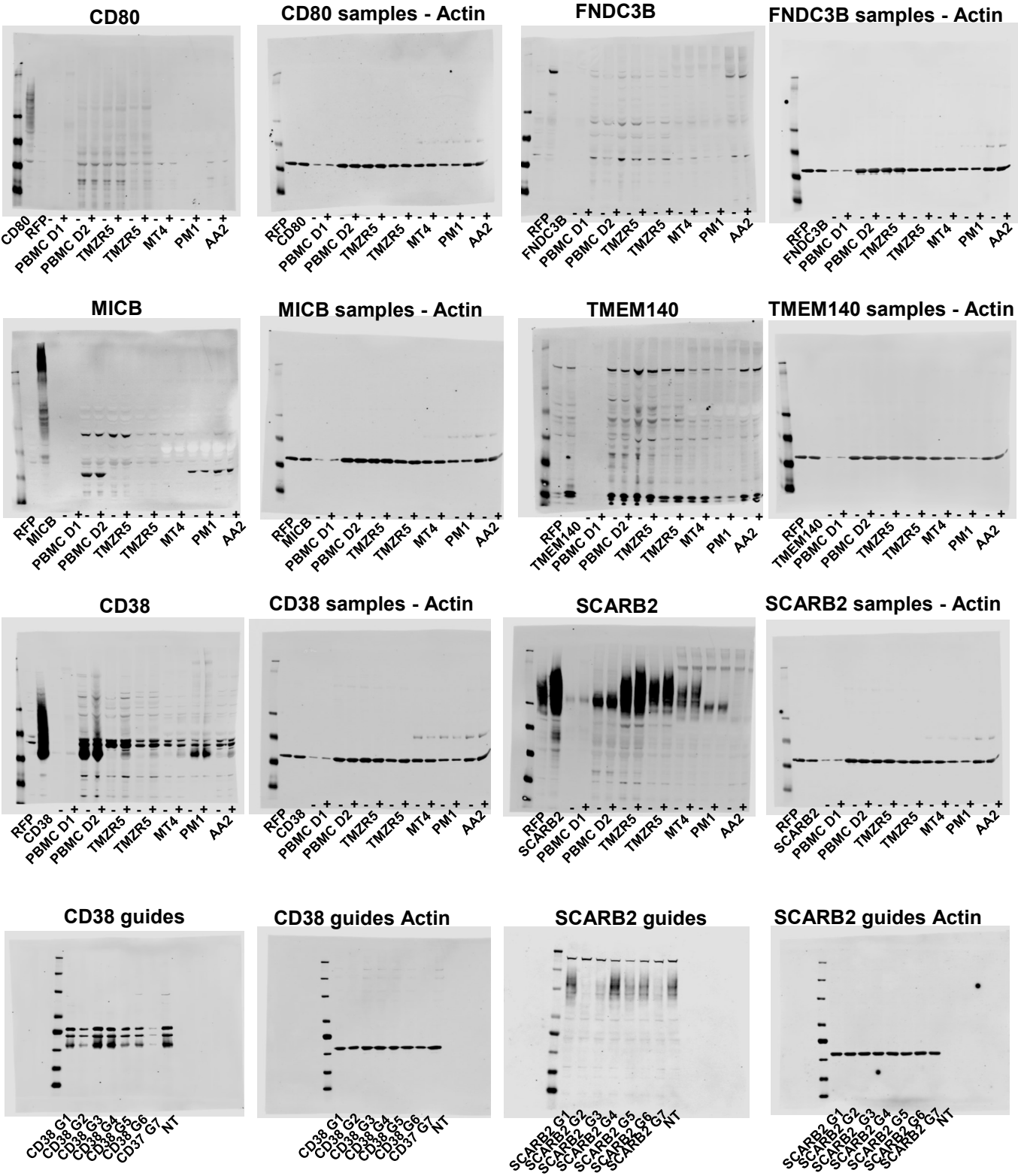

Supplement: S7 Fig — (PDF) [file ppat.1010973.s007.pdf]
